# Stabilization and destabilization of multimode solitons in nonlinear degenerate multi-pass cavities

**DOI:** 10.1038/s41377-026-02327-0

**Published:** 2026-06-26

**Authors:** Junhan Huang, Bingbing Zhu, Shanyue Li, Kun Ding, Zhensheng Tao

**Affiliations:** 1 State Key Laboratory of Surface Physics, Key Laboratory of Micro and Nano Photonic Structures (MOE), and Department of Physics, Fudan University, Shanghai, China; 2 Shanghai Key Laboratory of Metasurfaces for Light Manipulation, Fudan University, Shanghai, China

**Keywords:** Solitons, Nonlinear optics, Supercontinuum generation

## Abstract

Optical solitons in multimode nonlinear optical systems offer a unique platform for exploring the interplay of nonlinearity, dispersion, and spatial mode coupling, offering insights into complex nonlinear wave phenomena. Multi-pass cavities (MPCs) incorporating nonlinear Kerr media serve as prototypical systems, enabling high-efficiency supercontinuum generation and pulse compression. However, stabilizing femtosecond laser pulses in solid-medium-based MPCs (solid MPCs) under strong Kerr nonlinearity remains a significant challenge due to multimode coupling, which disrupts beam stability. In this work, we address this challenge by investigating the stability of laser pulses in MPCs using a Floquet and perturbative model. We identify novel mode-coupling-suppression (MCS) medium lengths, where destructive interference among multimode wave components suppresses coupling and facilitates soliton stabilization. Under MCS conditions, our simulations demonstrate stable beam propagation in solid MPCs with nonlinear phases up to 1.5π per pass, achieving >13-fold pulse compression with excellent spatio-spectral homogeneity. Our findings offer valuable guidance for designing advanced MPCs with tailored Kerr media.

## Introduction

Optical solitons—self-sustained, localized wave-packets stabilized by a balance between dispersion and nonlinearity—have been extensively studied due to their crucial role in high-speed optical communication^1^, ultrafast pulse generation^2,3^, and other nonlinear optical phenomena^4^. Of particular interest are multimode solitons, which involve interactions among multiple spatial modes, and exhibit rich spatiotemporal dynamics such as spatial self-organization^5–7^, soliton fission^8,9^, and the emergence of spatiotemporal solitons, also known as “light bullets”^10–13^.

**Fig. 1:**
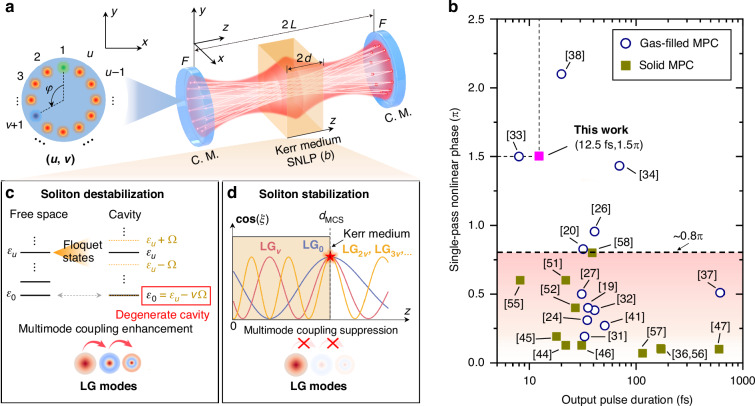
Mechanisms of stabilization and destabilization of multimode solitons in nonlinear MPCs. **a** Schematic of a nonlinear MPC geometry. C.M.: Concave mirror. Inset: Laser-spot distribution on a Herriott-type MPC with a degeneracy defined by indices (*u*, *v*). The green spot marks the initial position where the incident laser beam strikes, while the blue spot indicates its position after the first roundtrip, with the angular increment *φ* indicated. **b** Summary of state-of-the-art nonlinear MPCs for supercontinuum generation and pulse compression. The dashed line indicates the SNLP limit of existing solid MPCs. **c**, **d** Mechanisms underlying the destabilization and stabilization of multimode solitons in nonlinear MPCs

While multimode solitons have been extensively studied in optical fibers^14–16^, multi-pass cavities (MPCs) offer a complementary platform with weak transverse mode confinement and exceptional potential for high-energy applications^17,18^ (Fig. 1a). By circulating femtosecond laser pulses, nonlinear MPCs enable substantial spectral broadening, leading to high-throughput, and high-quality pulse compression with high power^19,20^, high pulse energy^20^, and few-cycle durations^21,22^. Commonly used Kerr media include noble gases^19–21,23–42^, and single^22,43–53^ or multiple^54–58^ solid media. Furthermore, MPCs allow access to diverse nonlinear regimes by precisely controlling dispersion through cavity mirrors or varying the nonlinear optical media.

A central design challenge in nonlinear MPCs is the long optical path length required to sustain the large number of roundtrips needed for spectral broadening, typically on the order of 30–50 passes. The large number of roundtrips demands exceptionally efficient broadband mirror coatings, which are costly, while the resulting total optical path length complicates pump–probe synchronization and increases long-term mechanical and thermal instability. These practical constraints ultimately stem from the intrinsically low single-pass nonlinear phase (SNLP) that must be maintained to preserve high spatio-spectral beam quality during nonlinear spectral broadening.

This limitation is particularly evident in solid-medium-based MPCs (solid MPCs), where strong nonlinearity readily induces spatio-spectral degradation, limiting operation to SNLP values below ~0.8π. In contrast, gas-filled MPCs can tolerate much higher SNLPs – up to ~2π (Fig. 1b). Despite its practical importance, the physical origin of this striking performance gap between solid and gas-filled MPCs remains poorly understood. Fundamentally, resolving this question is equivalent to identifying stable soliton solutions in MPCs operating under strong nonlinearity.

Previous theoretical studies have highlighted the role of multimode coupling in spatial degradation, both in single-pass setups^59^ and gas-filled MPCs^60,61^. Coupled-mode theory has shown that degenerate cavity geometries—where higher-order modes are phase-matched with the fundamental modes—strongly influence beam stability^61,62^. This insight is particularly important, as most nonlinear MPC implementations deliberately operate in such degenerate, *q*-preserving configurations to preserve the beam parameter *q* between the input and output of the cavity^63,64^. In addition, soliton-like dynamics in pulse propagation have been observed in solid MPCs at mid-infrared wavelengths^43^. Despite these insights, the underlying mechanisms that stabilize or destabilize multimode solitons remain elusive.

In this work, we theoretically investigate laser-beam stability in nonlinear MPCs with strong Kerr nonlinearity from the perspective of multimode solitons. Using Floquet analysis and first-order perturbation theory, we elucidate the mechanisms by which multimode coupling, induced by the interplay between cavity degeneracy and Kerr nonlinearity, destabilizes solitons (Fig. 1c). These analytical insights are supported by full numerical simulations that incorporate space-time coupling effects. Our results clarify the observed differences between gas-filled and solid MPCs. More importantly, we identify a novel concept of *mode-coupling-suppression (MCS) medium lengths* (*d*_MCS_), where destructive interference among multimode wave components within the Kerr medium suppresses multimode coupling. This stabilization mechanism facilitates soliton formation in degenerate nonlinear MPCs under high nonlinearity (Fig. 1d), with gas-filled MPCs emerging as a special case of the MCS condition.

Under the MCS condition, we demonstrate stable soliton propagation with a nonlinear phase up to *b* = 1.50 π per pass in solid MPCs (pink solid square in Fig. 1b), resulting in single-stage, >13-fold pulse compression with excellent spatio-spectral quality requiring only 9 roundtrips. This result exceeds the SNLP limits of the existing solid MPCs, offering valuable insights for designing advanced nonlinear MPCs with tailored Kerr media.

## Cavity degeneracy and beam-propagation stability

The conceptual schematic of a nonlinear MPC is shown in Fig. 1a. It consists of two concave mirrors (C.M.), each with a focal length *F*, separated by a distance of 2 *L*. At the cavity center, a Kerr medium with a length of 2 *d* interacts with femtosecond laser pulses, inducing an SNLP denoted as *b*. Here, SNLP is characterized by

1 $$b=\frac{2{\rm{\pi }}}{{\lambda }_{0}}{n}_{2}{\int }_{-d}^{d}I\left(z\right){dz}$$

where *λ*_0_ is the laser wavelength, *n*_2_ is the nonlinear refractive index, *z* is the coordinate along the cavity axis (with the origin at the cavity center), and *I*(*z*) is the peak laser intensity within the Kerr medium.

To investigate the stability of laser-beam propagation in nonlinear MPCs, we perform full space-time-coupled nonlinear Schrödinger equation (NLSE) simulations^65^ over a broad range of cavity parameters (see Methods). The numerical accuracy of the simulations is benchmarked against the spectral measurements and transform-limited (TL) pulse durations reported in previous experimental studies (see Supplementary Section S1).

In all the simulations, the input pulse has a duration of *τ*_*p*_ = 170 fs and a center-wavelength of *λ*_0_ = 1030 nm, representative of a typical Yb:KGW femtosecond laser. For each cavity geometry, the input beam profile is initialized as a mode-matched eigenmode of the corresponding linear cavity. Specifically, we use a Laguerre-Gaussian LG_0_ (Gaussian) mode with a beam waist of ${w}_{0}=\sqrt{\frac{{\lambda }_{0}{L}_{{\rm{eff}}}}{{\rm{\pi }}}}{\left(\frac{2F}{{L}_{{\rm{eff}}}}-1\right)}^{\tfrac{1}{4}}$, where ${L}_{{\rm{eff}}}=\left(L-d\right)+\frac{d}{{n}_{0}}$ is the effective cavity length, and *n*_0_ is the linear refractive index of the Kerr medium. Because only LG modes with zero azimuthal index (*l* = 0) are considered in this work, we label the modes solely by their radial index (*p*) (denoted as LG_*p*_).

The strength of SNLP *b* is adjusted by varying the input pulse energy *E*_0_:

2 $$b=\frac{8{n}_{2}{d}_{{\rm{eff}}}}{{w}_{0}^{2}{\lambda }_{0}}\frac{{E}_{0}}{{\tau }_{p}}$$

Here, ${d}_{{\rm{eff}}}={z}_{0,{\rm{Kerr}}}\arctan \left(\frac{d}{{z}_{0,{\rm{Kerr}}}}\right)$ represents an effective medium length, where ${z}_{0,{\rm{Kerr}}}={n}_{0}{L}_{{\rm{eff}}}\sqrt{\frac{2F}{{L}_{{\rm{eff}}}}-1}$ is the effective Rayleigh length. Additional details of the NLSE implementations are provided in Methods.

**Fig. 2:**
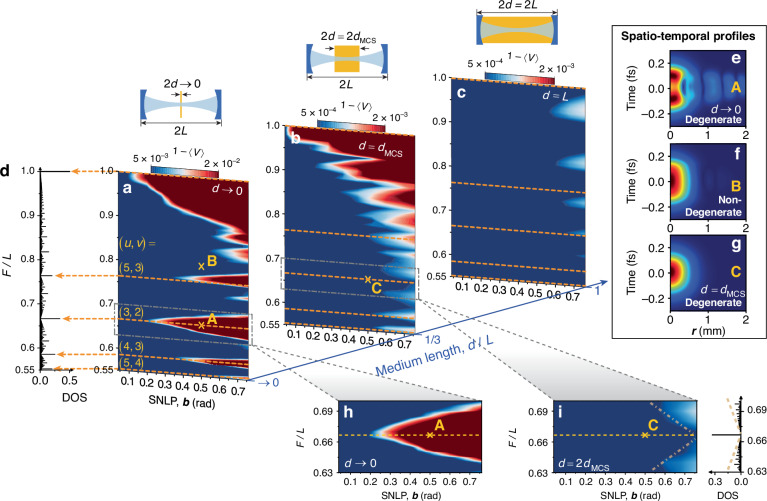
Stabilization landscape of nonlinear MPCs. Phase diagrams of output beam inhomogeneity as a function of cavity geometry *F*/*L* and SNLP *b*, for medium length corresponding to (**a**) a thin plate ($2d\to 0$), (**b**) 2 *d* = $\frac{2}{3}L$, and (**c**) a gas-filled MPC (2 *d* = 2 *L*). A unit refractive index (*n*_0_ = 1) is assumed. Conditions of A, B, and C are labeled. Dashed lines indicate degeneracy geometries with (*u*, *v*)=(5, 3), (3, 2), (4, 3), and (5, 4). **d** DOS as a function of *F*/*L*, with the correspondence of DOS peaks and inhomogeneity regions in (**a**) indicated by arrows. **e**–**g** Spatio-temporal profiles of the output beams after 10 roundtrips for Conditions A, B, and C, as labeled in (**a**–**c**). **h**, **i** Zoomed-in views of the phase diagrams near the degeneracy point (*u*, *v*)=(3, 2), as illustrated by the dashed-dotted boxes in (**a**, **b**). DOS results for the same *F*/*L* region are shown in the right panel

It is worth noting that when the refractive index deviates from unity (${n}_{0}\ne 1$), variations in the medium length 2 *d* also modify the cavity degeneracy, thereby breaking the *q*-preserving condition. In practice, this effect must be compensated by adjusting the cavity geometry, for example, by tuning *L* or *F* to restore degeneracy. To elucidate the fundamental role of cavity degeneracy in beam-propagation stability, the simulations shown in Fig. 2a–c therefore consider an idealized case in which the Kerr medium has a unit refractive index (*n*_0_ = 1). Under this assumption, the cavity degeneracy condition remains unchanged as the medium length 2 *d* is varied. Simulations incorporating realistic material parameters are presented later.

In all cases, the total cavity length is fixed at 2 *L* = 79.5 cm, while the mirror focal length *F* and medium length 2 *d* are varied. When $d/L\to 0$, implemented by setting 2 *d* = 1 mm (Fig. 2a), the simulation represents typical conditions of solid MPCs, where the medium length is much smaller than the cavity length. In contrast, *d*/*L* = 1.0 (Fig. 2c) represents a gas-filled MPC, where the gas medium occupies the entire cavity volume.

For the simulations using thin Kerr media (Fig. 2a), achieving large *b* values requires laser peak powers (${P}_{0}={E}_{0}/{\tau }_{p}$) exceeding the material critical power *P*_cr_, reaching up to *P*_0_/*P*_cr_ ≈ 15. Although such powers can induce self-focusing, the short medium length ensures the focal point lies outside the medium, preventing beam collapse and material damage in practice^66–68^. For thicker media (Fig. 2b, c), the peak power is typically below 50% of the critical power, which is sufficient to achieve the targeted *b* values.

We quantitatively assess beam instability by evaluating the spatio-spectral homogeneity ($\left\langle V\right\rangle$) of the output beam^57^ (see Methods). Figure 2a–c presents the two-dimensional plots of output-beam inhomogeneity ($1-\left\langle V\right\rangle$) after 10 roundtrips as functions of *b* and *F*/*L* for different *d*/*L* values.

Several key observations can be made. First, the spatio-spectral homogeneity improves systematically as the normalized medium length *d*/*L* increases, as seen by comparing Fig. 2a–c. Second, in the limit $d/L\to 0$ (Fig. 2a), cavities operating close to degeneracy exhibit pronounced spatio-spectral inhomogeneity.

To interpret these trends, it is essential to clarify what constitutes a degenerate cavity. For a practical Herriott-type MPC^69^, the *q*-preserving configuration results in closed ray trajectories, where an injected ray retraces its path after *u* roundtrips (*u* is an integer) and exits the cavity at the same position as the entrance point^62,63^. As a result, the circulating beam forms *u* discrete spots on the cavity mirrors (inset of Fig. 1a). This re-entrant condition indicates that the transverse ray positions advance periodically in both the *x* and *y* directions, with a fixed angular increment φ per roundtrip (inset of Fig. 1a). If the incident beam initially hits spot #1 (green spot), and the position reached after the first roundtrip corresponds to spot #(*v* *+* *1)* (blue spot), the angular increment is then given by $\varphi =2{\rm{\pi }}\frac{v}{u}$, where (*u*, *v*) are a pair of coprime integers with *v* < *u*.

It can be shown that this angular increment is equal to the Gouy phase shift accumulated per roundtrip, *φ*=*ξ*^RT^. For a symmetric MPC with a Kerr-medium length of 2 *d*, the roundtrip Gouy phase shift is

3 $${\xi }^{{\rm{RT}}}=4\arctan \left(\frac{1}{\sqrt{2F/{L}_{{\rm{eff}}}-1}}\right)$$

Equating the two expressions yields the following degeneracy condition:

4 $$4u\arctan \left(\frac{1}{\sqrt{2F/{L}_{{\rm{eff}}}-1}}\right)=2v{\rm{\pi }}$$

where the pair of coprime integers (*u*, *v*) uniquely specifies the cavity degeneracy. A detailed derivation is provided in Methods. Physically, Eq. (4) also indicates that the single-pass Gouy phase difference between the LG_*u*_ and LG_0_ modes (left-hand side) equals 2*v*π. Under this condition, all the LG modes whose radial index is an integer multiple of *u* also satisfy the degeneracy condition with the LG_0_ mode.

Quantitatively, the degree of cavity degeneracy can be characterized by a normalized density of states (DOS), which counts the number of LG modes that are degenerate with the LG_0_ mode (see Methods). A clear correlation is observed between the DOS peaks assuming *n*_0_ = 1 (Fig. 2d) and spatio-spectral inhomogeneity patterns (Fig. 2a), with enhanced inhomogeneity occurring at higher DOS. This observation is further corroborated by analyzing the spatio-temporal profiles for both degenerate (condition A) and non-degenerate (condition B) cases (Fig. 2e, f). Under the degenerate conditions, higher-order modes and pulse splitting are evident (Fig. 2e), indicating spatio-temporal instability.

Lastly, but more interestingly, we find that, at specific medium lengths, beam quality can be significantly improved even under the degenerate conditions. For instance, when $2d=\frac{2}{3}L$ with *F*/*L* = 2/3 and degenerate indices (*u*, *v*)=(3, 2) (condition C in Fig. 2b), the strong inhomogeneity observed in condition A (Fig. 2a) is remarkably suppressed, resulting in a spatio-spectrally homogeneous output beam. This medium length is denoted as *d*_MCS_ for the degeneracy indices (*u*, *v*)=(3, 2). The comparison between Fig. 2g, e further demonstrates that the spatio-temporal breakdown is mitigated under 2 *d* = 2*d*_MCS_.

To highlight this intriguing behavior, we further compare the zoomed-in views around the conditions A and C (Fig. 2h, i). Clearly, at 2 *d* = 2*d*_MCS_, the inhomogeneity at the degenerate point is sharply suppressed, while the surrounding regions with lower DOS exhibit some inhomogeneity, forming a “V”-shape pattern around the degenerate point (Fig. 2i). Importantly, Supplementary Fig. S15 presents the corresponding phase diagram for Fig. 2b using a realistic refractive index of *n*_0_ = 1.45. The same suppression of inhomogeneity at the degenerate point is observed, demonstrating that this effect is robust against variations in the medium refractive index. The high-homogeneity regions in Fig. 2a–c indicate the formation of discrete spatial solitons, where laser beams maintain stable spatial profiles within the nonlinear medium or on the cavity mirrors (see Supplementary Section S2).

## Floquet and perturbative model analysis

While the numerical simulations offer valuable insights into the stability landscape of discrete spatial solitons in nonlinear MPCs, the underlying mechanisms remain intricate. To elucidate these mechanisms, we employ analytical approaches based on Floquet and perturbative theories, which neglect space-time coupling effects.

Given that an optical cavity represents the periodic propagation of a light beam in space, its behavior can be described using Floquet theory^70^. In the linear regime, the system is governed by the Floquet eigenequation:

5 $$\left[{H}_{0}\left(r,z\right)-i\frac{\partial }{\partial z}\right]\left|{\Phi }_{n,m}\left(r,z\right){{\rangle }}={\varepsilon }_{n,m}\right|{\Phi }_{n,m}\left(r,z\right){{\rangle }}$$

as derived in Methods, where *H*_0_ is the linear-cavity Hamiltonian, $|{\Phi }_{n,m}\left(r,z\right){\rm{\rangle }}$ is the Floquet eigenmode, and ${\varepsilon }_{n,m}={\varepsilon }_{n}-m\Omega$ is the Floquet eigenvalue associated with the *m*-th replica of the LG_*n*_ mode. Here, $\Omega ={\rm{\pi }}/L$ denotes the Floquet “driving frequency”, and ${\varepsilon }_{n}={\xi }_{n}/\left(2L\right)$ is the eigenvalue of the LG_*n*_ mode, determined by its single-pass Gouy phase shift ${\xi }_{n}$.

In the Floquet framework, cavity degeneracy occurs when the eigenvalue of the (*n*, *m*)-th state coincides with that of the LG_0_ mode (Fig. 1c), i.e., ${\varepsilon }_{n,m}={\varepsilon }_{\mathrm{0,0}}$, or equivalently when the Gouy phase difference satisfies ${\xi }_{n}-{\xi }_{0}=2m{\rm{\pi }}$. This condition is equivalent to the degeneracy relation given by Eq. (4) when the mode indices (*n*, *m*) are integer multiples of the degeneracy indices (*u*, *v*). In other words, a given pair of degeneracy indices (*u*, *v*) supports an infinite set of Floquet eigenmodes $|{\Phi}_{n,m}\left(r,z\right){\rangle}$ that are degenerate with the LG_0_ mode.

To analyze a nonlinear MPC, we apply perturbation theory by expanding the nonlinear eigenmode in the Floquet basis: $|{\Psi}_{\mathrm{0,0}}{\rangle}={\sum}_{n,m}{C}_{n,m}|{\Phi}_{n,m}{\rangle}$, where the expansion coefficients *C*_*n,m*_ are given by

6 $${C}_{n,m}=\frac{-b{C}_{0,0}{|{C}_{0,0}|}^{2}{\Theta }_{n,m}\left(d\right)}{{\varepsilon }_{0,0}-{\varepsilon }_{n,m}}$$

The overlap integral ${\Theta }_{n,m}\left(d\right)$ in Eq. (6) is defined as

7 $${\Theta }_{n,m}\left(d\right)=\frac{1}{2{d}_{{\rm{eff}}}}\left\langle {\Phi }_{n,m}{|S}\left(z,d\right){\left|{\Phi }_{0,0}\right|}^{2}|{\Phi }_{0,0}\right\rangle$$

where *S*(*z*, *d*) is a periodic Heaviside function defining the Kerr-medium regions. Within the Floquet framework, the expansion coefficient of the LG_*n*_ mode is given by ${c}_{n}=\mathop{\sum }\nolimits_{m}{C}_{n,m}$. A detailed derivation is provided in Methods. Although the nonlinear eigenmodes can also be computed by numerically solving the simplified NLSE – neglecting space-time coupling [Eq. (20) in Methods] using the Fox-Li iteration algorithm^68,71^, the perturbative approach provides clearer analytical insight into the fundamental mechanisms governing soliton stabilization and destabilization in nonlinear MPCs.

**Fig. 3:**
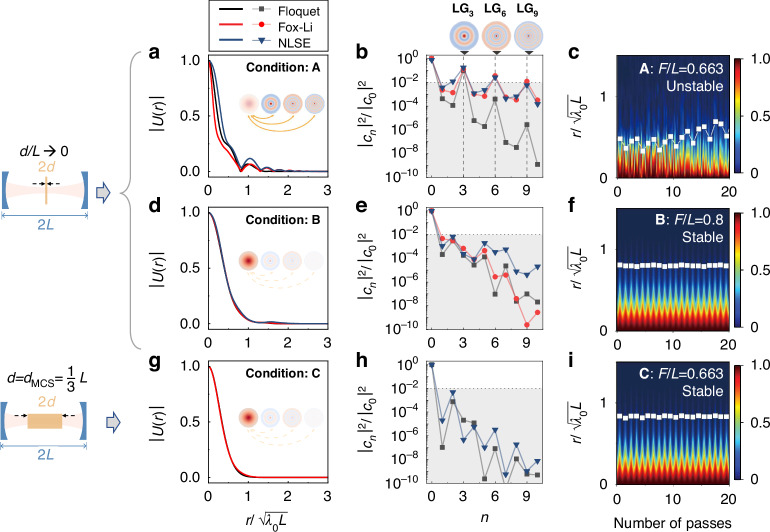
Stabilization and destabilization of multimode solitons. **a** Radial profiles of the eigenmode for a nearly degenerate solid MPC with *F*/*L* = 0.663 (close to Condition A) with SNLP *b* = 0.5, obtained from Floquet and perturbative analysis (black line), the Fox-Li algorithm (red line), and the full NLSE simulations (blue line). Inset: Illustration of energy transfer from the LG_0_ mode to higher-order LG modes. **b** Normalized expansion coefficients ${\left|{c}_{n}\right|}^{2}/{\left|{c}_{0}\right|}^{2}$ of the eigenmodes shown in (**a**). The horizontal dashed line indicates the amplitude of ${\left|{c}_{n}\right|}^{2}$ being 1% of the intensity of the fundamental mode ${\left|{c}_{0}\right|}^{2}$. The LG modes of LG_3_, LG_6_, and LG_9_ are labeled. **c** Beam-profile evolution of the eigenmode shown in (**a**), with the beam radii on a cavity mirror labeled by symbols. **d**–**f** Same as (**a**–**c**), but for the results at Condition B. **g**–**i** Same as (**a**–**c**), but for the results near Condition C

Figure 3a–f shows the eigenmode radial profiles for $d/L\to 0$ at *b* = 0.5 rad in degenerate and non-degenerate MPCs, while the results for the MCS condition (2 *d* = 2*d*_MCS_) are shown in Fig. 3g–i. These results correspond to the conditions A, B, and C in Fig. 2a–c. For consistency, a unit refractive index (*n*_0_ = 1) is assumed.

For a nearly degenerate solid MPC with *F*/*L* = 0.663 [close to the condition A with *F*/*L* = 2/3 and (*u*, *v*)=(3, 2)], the eigenmode radial profile $({|U}\left(r\right)|)$ deviates from a Gaussian shape, exhibiting ripples at large radii (Fig. 3a). Spatial-mode decomposition reveals clear signatures of multimode coupling: the LG_3_, LG_6_, and LG_9_ modes contribute significantly, whereas other higher-order modes remain suppressed (Fig. 3b). This modal structure can be understood by considering the Gouy phase relation in Eq. (4). For a degenerate cavity with (*u*, *v*)=(3, 2) and *n*_0_ = 1, the Gouy phase shift is $2\arctan \left(\frac{1}{\sqrt{2F/L-1}}\right)=\frac{2{\rm{\pi }}}{3}$, such that only the LG modes with a radial index *p* = 3*j* satisfy the degeneracy condition ξ_3*j*_ - ξ_0_ = 4*j*π (with *j* an integer) and are therefore degenerate with the LG_0_ mode.

To validate the perturbative approach, we compare the model predictions with eigenmodes obtained from Fox-Li iteration simulations^68,71^ (red lines and symbols; see Supplementary Section S3) and with full NLSE simulations with space-time-coupling effects considered (blue lines and symbols). For the NLSE results, the modal weights of the higher-order modes are extracted through a transverse-mode decomposition of the simulation results, with the fluence integrated over time. As shown in Fig. 3a, b, both numerical approaches reproduce the emergence and correct indexing of the higher-order modes. Although discrepancies in modal amplitudes appear at large *b*, the overall agreement confirms that the perturbation framework captures the essential physics governing multimode coupling in degenerate MPCs.

Further stability analysis (see Supplementary Section S4) shows that multimode coupling in degenerate solid MPCs significantly disrupts stable propagation of the eigenmode (Fig. 3c), indicating that cavity degeneracy hinders soliton stabilization.

In contrast, under the non-degenerate condition B (*F*/*L* = 0.8), the eigenmode maintains a nearly Gaussian profile (Fig. 3d), with the expansion coefficients showing negligible contributions from higher-order modes (Fig. 3e). The eigenmode exhibits stable discrete-soliton modes in the non-degenerate solid MPC (Fig. 3f).

The stark contrast between the degenerate and non-degenerate MPCs (Fig. 3a–f) is consistent with the phase diagram shown in Fig. 2a and can be explained by Eq. (6). When $d/L\to 0$, the overlap integral Θ_*n,m*_ is typically non-zero. Consequently, the degenerate condition (${\varepsilon }_{\mathrm{0,0}}={\varepsilon }_{n,m}$) causes the expansion coefficient *C*_*n,m*_ to diverge, driving substantial energy transfer from the LG_0_ mode to higher-order modes. We note that, while previous studies have revealed the importance of multimode coupling in degrading beam quality in degenerate MPCs^61,62^, our results provide a novel perspective based on Floquet and perturbative analysis.

## Mechanism of MCS medium length

For condition C, where the MPC is nearly degenerate and the medium length satisfies $2d={2d}_{{\rm{MCS}}}=\frac{2}{3}L$, Floquet and perturbative analysis shows that multimode coupling is effectively suppressed (Fig. 3g, h). Under this condition, the cavity eigenmode forms a soliton that propagates stably within the nonlinear MPC (Fig. 3i), in agreement with the full NLSE simulations shown in Fig. 2b.

The origin of this behavior can be understood by considering a strictly degenerate cavity, in which the denominator in Eq. (6) vanishes because ${\varepsilon }_{\mathrm{0,0}}-{\varepsilon }_{n,m}=0$. As a result, multimode coupling can be suppressed *only when* the overlap integral in the numerator equals zero, i.e., Θ_*n,m*_(*d*)=0. We refer to the corresponding medium length as the MCS medium length, *d*_MCS_.

For a mode $|{\Phi }_{n,m}\rangle$ that is degenerate with LG_0_, the overlap integral Θ_*n,m*_(*d*) in Eq. (7) can be calculated analytically. Imposing Θ_*n,m*_(*d*) = 0, we obtain

8 $$4u\arctan \left(\frac{d/{L}_{{\rm{eff}}}}{{n}_{0}\sqrt{2F/{L}_{{\rm{eff}}}-1}}\right)=2k{\rm{\pi }},k=1,2,\cdots ,v$$

which defines the MCS condition. The left-hand side corresponds to the accumulated Gouy phase difference within the Kerr medium between the LG_*u*_ and LG_0_ modes. Solving Eq. (8) determines the MCS medium length *d* = *d*_MCS_. A detailed derivation is provided in Supplementary Section S5.

**Fig. 4:**
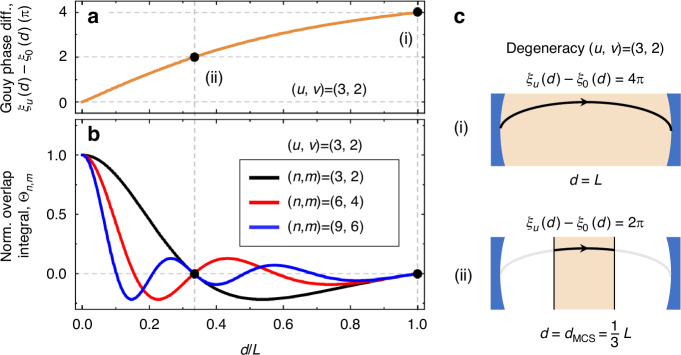
Illustration of MCS conditions. **a** Gouy phase difference accumulated over medium length 2 *d* between the LG_*u*_ and LG_0_ modes and (**b**) Corresponding normalized overlap integral Θ_*n*,*m*_ as a function of *d*/*L*, calculated for (*u*, *v*)=(3, 2) assuming *n*_0_ = 1. Dashed lines indicate medium lengths ($d=\frac{1}{3}L$) where the accumulated Gouy phase difference equals integer multiples of 2π, resulting in Θ_*n*,*m*_ = 0 for all degenerate eigenmodes. **c** Schematic illustration of the MCS conditions showing how discrete medium lengths yield integer multiples of 2π in the accumulated Gouy phase difference

To elucidate how multimode coupling is suppressed when *d* = *d*_MCS_, we plot the Gouy phase differences given by the left-hand side of Eq. (8) and the corresponding overlap integral Θ_*n*,*m*_(*d*) in Fig. 4a, b, respectively. Following conditions A and C in Fig. 2a, b, we consider cavity degeneracy of (*u*, *v*)=(3, 2) and assume a unit refractive index (*n*_0_ = 1) for the Kerr medium. Under these conditions, Eq. (8) reduces to $4u\arctan \left(\frac{d/L}{\sqrt{2F/L-1}}\right)=2k{\rm{\pi }}$.

As shown in Fig. 4a, when the Kerr medium occupies the entire cavity (*d* = *L*), the Gouy phase difference equals 2*v*π (i.e., 4π for *v* = 2). Consequently, the overlap integral Θ_*n,m*_ vanishes for all degenerate eigenmodes with (*n*, *m*)=(3, 2), (6, 4), (9, 6), …. (Fig. 4b). This result indicates that gas-filled MPCs can be regarded as a special case of the MCS condition, explaining their ability to support high-quality beam propagation and efficient nonlinear light-matter interactions.

Equation (8) further indicates that the medium length can be reduced such that the accumulated Gouy phase difference within the Kerr medium equals 2π, which also makes Θ_*n,m*_ = 0 (Fig. 4a–b). This corresponds to an MCS medium length of ${d}_{{\rm{MCS}}}=\frac{1}{3}L$ (Fig. 4c), in excellent agreement with the numerical results shown in Fig. 2b and Fig. 3g–i. Taken together, these results indicate that Eq. (8) represents a destructive-interference condition among multimode components within the Kerr medium, driving Θ_*n,m*_→0 and consequently suppressing the coefficients *C*_*n*,*m*_.

While the above analysis focuses on degenerate nonlinear MPCs, the Floquet and perturbative model can be generalized to arbitrary MPC geometries by numerically calculating Θ_*n*,*m*_ (see Supplementary Section S5). In Supplementary Fig. S3, we present phase diagrams computed assuming *n*_0_ = 1. The key features observed in the full NLSE simulations (Fig. 2a–c) can be well reproduced, including (i) the strong correlation between beam instability and degeneracy points in the limit $d/L\to 0$, and (ii) the recovery of beam quality at 2 *d* = 2 *d*_MCS_. The close agreement with the NLSE results indicates that, despite neglecting space-time coupling, the analytical model provides a robust framework for understanding the mechanisms governing the observed phase diagrams.

Beyond understanding the main features, space-time coupling indeed introduces secondary effects that refine the stability landscape. One particular example is observed in gas-filled MPCs with *d*/*L* = 1, where spatio-spectral inhomogeneity emerges at high nonlinearity even slightly away from the exact degenerate points (Fig. 2c). A similar effect is visible in solid MPCs when comparing Fig. 2a and Supplementary Fig. S3a. These deviations arise because the space-time coupling perturbs the ideal destructive-interference condition for Θ_*n*,*m*_ = 0, thereby re-introducing multimode coupling and degrading beam stability.

## Supercontinuum generation in MPCs operating at high nonlinearity

The identification of the MCS condition opens new possibilities for high-quality supercontinuum generation (SCG) and pulse compression in MPCs operating at high nonlinearity. In the preceding analysis, however, two simplifying assumptions were made to elucidate the underlying mechanisms. First, the Kerr medium was assumed to have a unit refractive index (*n*_0_ = 1), such that variations in the medium length 2*d* do not modify the cavity degeneracy. Second, the analysis focused on degenerate conditions with relatively low indices (e.g., (*u*, *v*)=(3, 2)), which in practice permit only a limited number of roundtrips.

For practical MPC designs operating under the MCS condition, these assumptions must be relaxed. In particular, when ${n}_{0}\ne 1$, the cavity parameters *F*, *L*, and *d*_MCS_ become intrinsically coupled. In the following, we present concrete design strategy that incorporates realistic material properties and enables stable operation at high nonlinearity.

We consider an MPC with a total cavity length of 2 *L* = 25 cm and choose a higher-order degeneracy (*u*, *v*)= (11, 9), which allows up to 11 roundtrips. Selecting *k* = 6 yields an accumulated Gouy phase difference of 12π in Eq. (8). The resulting Gouy phase difference and overlap integral Θ_*n*,*m*_ are shown in Supplementary Fig. S13. Under these conditions, the MCS medium length is given by

9 $${d}_{{\rm{MCS}}}={n}_{0}{L}_{{\rm{eff}}}\frac{\tan \left(k{\rm{\pi }}/2u\right)}{\tan \left(v{\rm{\pi }}/2u\right)}$$

as derived in Methods. This expression yields 2*d*_MCS_ = 10.67 cm for the present design. The corresponding mirror focal length is then determined using Eq. (4) to be *F* = 5.88 cm. Alternatively, this procedure can be inverted to determine *d*_MCS_ and *L* for a given *F* (see Methods). All cavity geometry parameters are summarized in Table 1.

**Table 1:** Key parameters for the NLSE simulations

Cavity Geometry	
Cavity length, 2 *L* (cm)	25
Focal length, *F*(cm)	5.88
Medium length, 2 *d* (cm)	10.67
GDD at each cavity mirror (fs^2^)	−1970
Kerr Medium (Fused Silica)	
*n*_0_	1.45^76^
*n*_2_ (m^2^ W^-1^)	2.4 × 10^−20^ ^77^
Group velocity dispersion	Calculated using data in ref. ^76^
χ_K_	0.2^65^
τ_1_ (fs)	20^65^
τ_2_ (fs)	40^65^
Laser Parameters	
τ_p_ (fs)	170
λ_0_ (nm)	1030
*E*_0_ (μJ)	1.14
*I*_0_ (W cm^-2^)	3.8 × 10 ^10^

Fused silica is selected as the Kerr medium, with realistic material parameters implemented (Table 1). χ_K_, τ_1_, and τ_2_ are the Raman response coefficients (see Methods). Notably, because of the relatively thick medium length, material GDD must be accounted for. We therefore introduce a compensating negative GDD of -1970 fs^2^ per bounce on the cavity mirrors, following the approaches used in recent MPC studies^23,46^. The input laser pulses have a duration of τ_p_ = 170 fs and pulse energy of *E*_0_ = 1.14 μJ at λ_0_ = 1030 nm, corresponding to a peak intensity *I*_0_ of 3.8 ×10^10 ^W cm^-2^ and a SNLP of 1.5π.

**Fig. 5:**
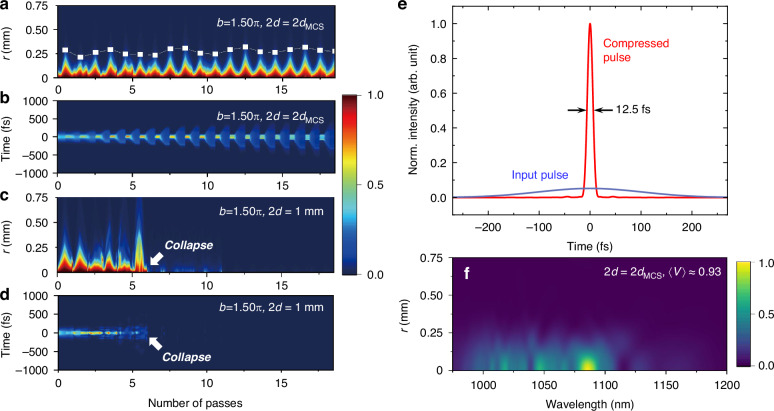
MCS-enabled supercontinuum generation and pulse compression. **a** Beam-profile evolution and (**b**) temporal pulse evolution obtained from NLSE simulations for femtosecond pulses in a degenerate cavity with (*u*, *v*)=(11, 9), operated at an SNLP of *b* = 1.5π and a medium length 2 *d* = 2 *d*_MCS_. Realistic material parameters are implemented in the simulations. Beam radii on a cavity mirror are labeled by symbols in (**a**). Transverse beam profiles are obtained by integrating optical fluence over time. **c**, **d** Corresponding simulations for the same cavity geometry and nonlinear phase, but using a thin-plate Kerr medium (2*d* = 1 mm). **e** Temporal profile of the compressed output pulse (red line), reconstructed from the simulated spectrum in (**a**, **b**), compared with the input pulse (blue line). **f** Radial distribution of the output pulse spectrum obtained from the simulations in (**a**, **b**)

Figure 5a shows the evolution of transverse beam profiles over 18 passes (9 roundtrips), demonstrating stable propagation. Figure 5b displays the corresponding evolution of the pulse temporal profiles. In each pass, the pulse experiences material-dispersion-induced broadening, which is compensated by the negative chirp provided by the cavity mirrors. Together, these results demonstrate that, under the MCS condition, both the spatial mode and the temporal profile remain stably preserved. For comparison, replacing the thick Kerr medium with a thin fused-silica plate (*d* = 1.0 mm), as commonly employed in solid MPCs, leads to beam collapse in both space and time after only 6 passes (Fig. 5c, d).

The spectrum bandwidth obtained under the MCS condition is sufficient for direct pulse compression from 170 fs to ~12.5 fs (Fig. 5e), achieving >13-fold compression in a single-stage, all-solid MPC compressor with only 9 roundtrips. Importantly, the spatio-spectral homogeneity remains as high as 0.93, even under such strong nonlinearity (Fig. 5f), confirming the effectiveness of the MCS condition in preserving beam quality.

## Critical power constraint

In addition to multimode coupling arising from cavity degeneracy, a second fundamental constraint on stable beam propagation under strong Kerr nonlinearity is imposed by the critical power for self-focusing, *P*_cr_, which limits the nonlinear phase that can be accumulated without inducing catastrophic beam collapse.

For MPCs with long medium lengths, this constraint has been analyzed in previous work^63^. By requiring the input peak power to satisfy *P*_0_<*P*_cr_, one obtains an upper bound on the SNLP, given by

10 $${b}_{\max }=4\arctan \left(\frac{d}{{z}_{0,{\rm{Kerr}}}}\right)$$

as derived in Methods. Equation (10) predicts a maximum SNLP approaching 2π for gas-filled MPCs with $d\gg {z}_{0,{\rm{Kerr}}}$. Physically, this indicates that for gas-filled MPCs, cavity nonlinearity is ultimately limited by the onset of self-focusing collapse.

However, this long-medium approximation no longer applies when the nonlinear medium is short. In this regime, self-focusing may occur outside the material, allowing substantially higher peak powers without collapse inside the medium, as recently demonstrated in multiplate geometries^66,68^. The appropriate physical requirement is therefore that the self-focusing length *z*_SF_ exceeds the medium length 2 *d*. Imposing *z*_SF_ > 2 *d* yields a modified upper bound on the SNLP,

11 $${b}_{\max }=4\arctan \left(\frac{d}{{z}_{0,{\rm{Kerr}}}}\right){\left[\sqrt{{\left(\frac{0.184}{d/{z}_{0,{\rm{Kerr}}}}\right)}^{2}+0.0219}+0.852\right]}^{2}$$

with the derivation provided in Methods. Notably, Eq. (11) smoothly converges to Eq. (10) in the limit $d/{z}_{0,{\rm{Kerr}}}\to {\infty }$ (Supplementary Fig. S4), confirming its consistency across regimes.

Supplementary Fig. S4 summarizes the constraints imposed by Eqs. (10–11) as a function of the cavity geometry, parameterized by $d/{z}_{0,{\rm{Kerr}}}$. Importantly, the critical power constraint alone *does not* fully determine *b*_max_, as it neglects the role of multimode coupling. When only self-focusing collapse is considered, thin Kerr media can, in principle, tolerate very high peak powers, allowing *b*_max_ to exceed 3π in the limit $d\to 0$. Full NLSE simulations and our Floquet-perturbative analysis, however, reveal a markedly different behavior: strong multimode coupling in this regime destabilizes the beam and precludes stable soliton formation. Therefore, our work qualitatively identifies an optimal intermediate regime for solid MPCs, in which increasing the medium length to satisfy the MCS condition suppresses multimode coupling, while retaining a sufficiently short nonlinear medium enables high input peak power.

Furthermore, we note that in the simulations shown in Fig. 5a–b, stable beam propagation is achieved at an SNLP of ~1.5π for $d/{z}_{0,{\rm{Kerr}}}\approx 1.154$, exceeding the limit predicted by Eq. (11) (Supplementary Fig. S4). This discrepancy can be attributed to two factors. First, Eq. (11) relies on an empirical estimate of the self-focusing length *z*_SF_ derived for quasi-continuous waves^72^, whereas material GDD is known to increase the self-focusing threshold for ultrashort pulses^73^. Second, Eq. (11) represents an idealized upper bound corresponding to an MPC with an infinite number of roundtrips. In practice, real MPCs support only a finite number of passes (e.g., 18 passes in Fig. 5a–b), allowing the experimentally accessible SNLP to exceed the ideal bound.

## Robustness of MCS condition

To evaluate the robustness of the MCS condition under realistic experimental conditions, we systematically examined a range of practical perturbations based on the simulations in Fig. 5a–b. These perturbations include cavity mirror losses of up to 3%, degraded incident beam quality with M^2^ values up to 1.8, beam divergence effects introduced by varying the beam waist by up to 40% relative to the cavity eigenmode, longitudinal offsets and length errors of the Kerr medium, and second- and higher-order mirror dispersion. In all cases, stable beam propagation and effective suppression of multimode coupling are preserved (see Supplementary Section S8), demonstrating that the MCS condition is robust against realistic experimental imperfections. Notably, under conditions slightly deviated from those in Fig. 5a, b, it is possible to obtain a sub-10-fs TL pulse duration with modest reduction in the spatio-spectral homogeneity $\left\langle V\right\rangle$, which may be favorable in applications prioritizing pulse compression.

At high incident laser powers, thermal lensing in the Kerr medium and cavity mirrors can modify the effective cavity geometry, thereby perturbing cavity degeneracy and compromising the MCS condition. Our analysis shows that the cavity degeneracy can be restored by adjusting the cavity length, rendering the MCS framework compatible with high-average-power operation (see Supplementary Section S9).

## Conclusions

Although our analysis underscores the superior performance of gas-filled MPCs, solid MPCs offer unique flexibility through the tailored design of Kerr media, as we understand the mechanism to suppress multimode coupling in nonlinear MPCs. While our study focuses on a single bulk Kerr medium placed at the cavity center, the same MCS mechanism can be extended to configurations involving multiple periodic or nonperiodic, centro-symmetric, or asymmetric distributions of Kerr media. Various MPC geometries beyond the Herriott-type configuration can also be explored. These advancements open up new possibilities for applications of optical cavities in SCG and other nonlinear processes.

## Methods

### NLSE simulations

Nonlinear MPCs are simulated using the equivalent-lens sequence model, where cavity mirrors are replaced by thin focusing lenses and the beam is assumed to propagate in the forward direction. The Kerr medium, with a thickness of 2 *d*, is placed at the cavity center. The forward-propagation NLSE with radial symmetry is given by^65^

12 $$\frac{\partial U}{\partial z}=\frac{i}{2{n}_{0}{k}_{0}}{{\mathcal{T}}}^{-1}{\nabla }_{\perp }^{2}U+i{\mathcal{D}}U+i\frac{{\omega }_{0}}{c}{n}_{2}{\mathcal{T}}\left[\left(1-{\chi }_{K}\right){\left|U\right|}^{2}+{\chi }_{K}{\int }_{-\infty }^{t}h\left(t-{t}^{{\prime} }\right){\left|U\left({t}^{{\prime} }\right)\right|}^{2}{{dt}}^{{{\prime} }}\right]U$$

where *U* is the complex field amplitude, *t* is the retarded time $t-z/{v}_{g}$, with *v*_*g*_ being the group velocity near the carrier frequency *ω*_0_, and *k*_0_ is the vacuum wavevector. The dispersion operator is ${\mathcal{D}}=\frac{{k}^{{\prime\prime} }}{2}{\left({i\partial }_{t}\right)}^{2}$. The operator ${\mathcal{T}}=\left(1+\frac{{i\partial }_{t}}{{\omega }_{0}}\right)$ accounts for self-steepening. The Raman response is parameterized by ${\chi }_{K}$ and the response function

13 $$h\left(t\right)=\frac{2}{3}\frac{{\tau }_{1}^{2}+{\tau }_{2}^{2}}{{\tau }_{1}{\tau }_{2}^{2}}{e}^{-t/{\tau }_{2}}\sin \left(t/{\tau }_{1}\right)$$

with *τ*_1_ and *τ*_2_ being the Raman time constants. The effect of the cavity mirrors is incorporated through the thin-lens transformation: ${U}^{{\prime} }=U{e}^{-i{\rm{\pi }}{r}^{2}/{\lambda }_{0}F}$.

The NLSE is numerically solved using the split-step Fourier method^74^. To account for large variations in beam radius during propagation, we implement a non-uniform radial grid defined by the transformation $r={r}_{0}\left({e}^{y}-1\right)$, with *y* uniformly discretized. Here, *r*_0_ is chosen to match the beam waist radius of the corresponding cavity eigenmode. Numerical accuracy is controlled by maintaining a local error of O(*dz*^3^), where *dz* is the propagation step size.

### Spatio-spectral inhomogeneity

Quantitatively, the spatio-spectral homogeneity can be characterized by the spectral overlap integral^57^ $V\left(r\right)=\frac{{\left\{{\int }_{\lambda }{\left[I\left({\rm{\lambda }},r\right)I\left({\rm{\lambda }},0\right)\right]}^{1/2}d{\rm{\lambda }}\right\}}^{2}}{{\int }_{\lambda }I\left({\rm{\lambda }},r\right)d\lambda \cdot {\int }_{{\rm{\lambda }}}I\left({\rm{\lambda }},0\right)d{\rm{\lambda }}}$, where *I*(λ,*r*) represents the spectral intensity of the output beam at radial coordinate *r*. The average overlap integral across the output beam is given by $\left\langle V\right\rangle =\frac{\int V(r)I(r){rdr}}{\int I(r){rdr}}$.

### Derivation of the linear-cavity degeneracy condition

We consider an MPC containing a Kerr medium of length 2 *d* and refractive index *n*_0_, positioned symmetrically at the center of the cavity. The regions outside the Kerr medium are assumed to be vacuum. The total cavity length is 2 *L*, and the cavity mirrors have focal length *F*.

The single-pass ABCD matrix of the MPC can be written as

$${\bf{M}}=\left(\begin{array}{cc}1 & d\\ 0 & 1\end{array}\right)\left(\begin{array}{cc}1 & 0\\ 0 & \frac{1}{{n}_{0}}\end{array}\right)\left(\begin{array}{cc}1 & L-d\\ 0 & 1\end{array}\right)\left(\begin{array}{cc}1 & 0\\ -\frac{1}{F} & 1\end{array}\right)\left(\begin{array}{cc}1 & L-d\\ 0 & 1\end{array}\right)\left(\begin{array}{cc}1 & 0\\ 0 & {n}_{0}\end{array}\right)\left(\begin{array}{cc}1 & d\\ 0 & 1\end{array}\right)$$

After straightforward matrix multiplication, this reduces to

14 $${\bf{M}}=\left(\begin{array}{cc}1-\frac{{L}_{{\rm{eff}}}}{F} & {n}_{0}{L}_{{\rm{eff}}}\left(2-\frac{{L}_{{\rm{eff}}}}{F}\right)\\ -\frac{1}{{n}_{0}F} & 1-\frac{{L}_{{\rm{eff}}}}{F}\end{array}\right)$$

where the effective cavity length in the presence of the Kerr medium is

15 $${L}_{{\rm{eff}}}=\left(L-d\right)+\frac{d}{{n}_{0}}$$

The roundtrip Gouy phase shift ${\xi }^{{\rm{RT}}}$ of the cavity eigenmode is determined by half the trace of the ABCD matrix,

16 $${\xi }^{{\rm{RT}}}=2\arccos \left(1-\frac{{L}_{{\rm{eff}}}}{F}\right)=4\arctan \left(\frac{1}{\sqrt{2F/{L}_{{\rm{eff}}}-1}}\right)$$

which is equivalent to Eq. (3) in the main text.

We next relate ${\xi }^{{\rm{RT}}}$ to the angular increment φ in a *q*-preserving MPC^63,69^. For such MPCs, the re-entrant condition requires that the angular increment satisfies $\varphi =2{\rm{\pi }}\frac{v}{u}$, where (*u*, *v*) are the cavity degeneracy indices. As shown in ref. ^69^, the cavity geometry satisfies

17 $$C\equiv \frac{{L}_{{\rm{eff}}}}{F}=1-\cos \left(\frac{\varphi }{2}\right)$$

Using the trigonometric identity $\sin \theta =\sqrt{1-{\cos }^{2}\theta }$, Eq. (17) yields $\sin \left(\frac{\varphi }{2}\right)=\sqrt{C\left(2-C\right)}$. Expressing the roundtrip Gouy phase shift in terms of *C*, we obtain

18 $${\xi }^{{\rm{RT}}}=4\arctan \left(\frac{C}{\sqrt{C\left(2-C\right)}}\right)=4\arctan \left(\frac{1-\cos \left(\varphi /2\right)}{\sin \left(\varphi /2\right)}\right)$$

Applying the half-angle identity $\tan \left(\frac{\varphi }{4}\right)=\frac{1-\cos \left(\varphi /2\right)}{\sin \left(\varphi /2\right)}$, we arrive at

19 $${\xi }^{{\rm{RT}}}=4\arctan \left[\tan \left(\frac{\varphi }{4}\right)\right]=\varphi$$

Finally, combining Eqs. (16) and (19) yields the linear-cavity degeneracy condition presented in Eq. (4) of the main text.

### Floquet theory

We begin with the simplified NLSE given by

20 $$i\frac{\partial U}{\partial z}=-\frac{1}{2{n}_{0}{k}_{0}}{\nabla }_{\perp }^{2}U+{V}_{l}\left(r,z\right)U+b{V}_{k}\left(r,z,U\right)U$$

where the cavity-mirror “potential” ${V}_{l}\left(r,z\right)$ is given by

21 $${V}_{l}\left(r,z\right)=\frac{\pi {r}^{2}}{{\lambda }_{0}F}\mathop{\sum }\limits_{n}\delta \left(z-\left(2n+1\right)L\right)$$

and the Kerr nonlinear term $b{V}_{k}\left(r,z,U\right)$ is

22 $$b{V}_{k}\left(r,z,U\right)=-{n}_{2}{k}_{0}{I}_{0}\left(z=0\right)S\left(z,d\right){{|U|}}^{2}\equiv -\frac{b}{2{d}_{{\rm{eff}}}}S\left(z,d\right){{|U|}}^{2}$$

Here, ${V}_{l}\left(r,z\right)$ represents the cavity-mirror focusing modeled using the thin-lens approximation. ${V}_{k}\left(r,z,U\right)$ describes the self-focusing effect induced by Kerr nonlinearity. $S\left(z,d\right)$ is a periodic Heaviside function defining the medium length:

23 $$S\left(z,d\right)\equiv \left\{\begin{array}{l}1,\left|z\right|\le d\\ 0,\mathrm{else}\end{array}\right.,z\in \left[-L,L\right],\mathrm{and}\,S\,\left(z+2L\right)=S\left(z\right)$$

For convenience, we define an effective length $2{d}_{{\rm{eff}}}=2{z}_{0,\mathrm{Kerr}}\arctan \left(d/{z}_{0,\mathrm{Kerr}}\right)$. The nonlinear phase per pass is then given by

24 $$b={n}_{2}{k}_{0}{I}_{0}\cdot {2d}_{\mathrm{eff}}$$

We use Floquet theory to analyze the linear contribution in Eq. (20). The linear Hamiltonian is given by ${H}_{0}\left(r,z\right)=-\frac{1}{2{n}_{0}{k}_{0}}{\nabla }_{\perp }^{2}+{V}_{l}\left(r,z\right)$, and the corresponding Floquet eigenequation is:

25 $$\left[H_{0}(r,z)-i\frac{\partial}{\partial z}\right]|{\Phi }_{n}\left(r,z\right){{\rangle }}={\varepsilon }_{n}|{\Phi }_{n}\left(r,z\right)\rangle$$

Since the Floquet Hamiltonian is periodic along *z*, its eigenmode $|{\Phi }_{n}\left(r,z\right)\rangle$ has an infinite number of replicas, $|{\Phi }_{n,m}\left(r,z\right)\rangle$, with Floquet eigenvalue ${\varepsilon }_{n,m}={\varepsilon }_{n}-m\Omega$, where $\Omega ={\rm{\pi }}/L$ is the “driving frequency”.

In the subspace with zero angular momentum, the Floquet state is expressed as

26 $$|{\Phi }_{n,m}\left(r,z\right)\rangle =\frac{\sqrt{2/\pi }}{w\left(z\right)}{L}_{n}\left[2\frac{{r}^{2}}{{w}^{2}\left(z\right)}\right]{e}^{-\frac{{r}^{2}}{{w}^{2}\left(z\right)}}{e}^{-{ik}\frac{{r}^{2}}{2R\left(z\right)}}{e}^{-i\frac{1}{2}{\xi }_{n}\left(z\right)}{e}^{i\frac{{\xi }_{n}\left(L\right)}{2L}z-{im}\Omega z}\equiv {\psi }_{n}\left(r,z\right){e}^{i\frac{{\xi }_{n}\left(L\right)}{2L}z-{im}\Omega z}$$

where ${\psi }_{n}\left(r,z\right)=\frac{\sqrt{2/\pi }}{w\left(z\right)}{L}_{n}\left[2\frac{{r}^{2}}{{w}^{2}\left(z\right)}\right]{e}^{-\frac{{r}^{2}}{{w}^{2}\left(z\right)}}{e}^{-{ik}\frac{{r}^{2}}{2R\left(z\right)}}{e}^{-i\frac{1}{2}{\xi }_{n}\left(z\right)}$ is the LG_n_ mode, with *L*_*n*_ representing the n-th order Laguerre polynomial, and ${\xi }_{n}\left(z\right)$ being the single-pass Gouy phase shift:

27 $${\xi }_{n}\left(z\right)=\left\{\begin{array}{c}2\left(2n+1\right)\arctan \left(\frac{z}{{z}_{0}}\right),z\le d\\ 2\left(2n+1\right)\arctan \left(\frac{z-d+d/{n}_{0}}{{z}_{0}}\right),z > d\end{array}\right.$$

with $${z}_{0}=\left\{\begin{array}{c}{n}_{0}{L}_{{\rm{eff}}}\sqrt{2F/{L}_{{\rm{eff}}}-1},z\le d\\ {L}_{{\rm{eff}}}\sqrt{2F/{L}_{{\rm{eff}}}-1},z > d\end{array}\right.$$ being the Rayleigh length of the linear-cavity eigenmodes. The accumulated Gouy phase upon one pass through the cavity is ${\xi }_{n}\left(L\right)$. The Floquet eigenvalue, thus, is given by:

28 $${\varepsilon }_{n,m}=\frac{{\xi }_{n}\left(L\right)}{2L}-m\Omega$$

Cavity degeneracy occurs when the eigenvalue of the (*n*, *m*)-th state coincides with that of the ground state, ${\varepsilon }_{n,m}={\varepsilon }_{\mathrm{0,0}}$, which yields the same degeneracy condition as shown in Eq. (4).

### Perturbation theory

We apply perturbation theory to derive the ground-state solitons in nonlinear MPCs. Since the soliton state shares the same periodicity as the cavity, the ground-state soliton $|{\Psi }_{\mathrm{0,0}}\left(r,z\right)\rangle$ and its eigenenergy ${\epsilon }_{\mathrm{0,0}}$ can be expanded as a superposition of the Floquet eigenmodes and eigenvalues:

29 $$|{\Psi }_{0,0}\left(r,z\right)\rangle ={C}_{0,0}|{\Phi }_{0,0}\left(r,z\right)\rangle +b\mathop{\sum }\limits_{{n}^{{\prime} }\ne 0,{m}^{{\prime} }\ne 0}{\widetilde{C}}_{{n}^{{\prime} },{m}^{{\prime} }}|{\Phi }_{{n}^{{\prime} },{m}^{{\prime} }}\left(r,z\right)\rangle$$

and

30 $${\epsilon }_{0,0}={\varepsilon }_{0,0}+b{\Delta }_{0,0}$$

Here, we introduce the perturbation terms by assuming $b\ll 1$. The nonlinear eigenequation is

31 $$\left[H\left(r,z\right)-i\frac{\partial }{\partial z}\right]|{\Psi }_{0,0}\left(r,z\right)\rangle ={\epsilon }_{0,0}|{\Psi }_{0,0}\left(r,z\right)\rangle$$

where the nonlinear Hamiltonian is $H\left(r,z\right)={H}_{0}\left(r,z\right)+b{V}_{k}\left(r,z,U\right)$. By inserting Eqs. (29–30) into Eq. (31), we obtain

32 $$\mathop{\sum }\limits_{{n}^{{\prime} }\ne 0,{m}^{{\prime} }\ne 0}\left({\varepsilon }_{0,0}-{\varepsilon }_{{n}^{{\prime} },{m}^{{\prime} }}\right)b{\widetilde{C}}_{{n}^{{\prime} },{m}^{{\prime} }}|{\Phi }_{{n}^{{\prime} },{m}^{{\prime} }}\rangle +b{\Delta }_{0,0}{C}_{0,0}|{\Phi }_{0,0}{{\rangle }}={V}_{k}{C}_{0,0}|{\Phi }_{0,0}\rangle$$

By taking the inner product with ${{\langle }}{\Phi }_{n,m}|$ and using the orthogonal relation of the Floquet eigenmodes, we arrive

33 $${\widetilde{C}}_{n,m}=\frac{{C}_{0,0}\langle {\Phi }_{n,m}|{V}_{k}|{\Phi }_{0,0}\rangle }{{\varepsilon }_{0,0}-{\varepsilon }_{n,m}}$$

We further evaluate the overlap integral:

34 $$\langle {\Phi }_{n,m}|{V}_{k}|{\Phi }_{0,0}\rangle =-\frac{1}{2{d}_{{\rm{eff}}}}{|{C}_{0,0}|}^{2}\langle {\Phi }_{n,m}{|S}\left(z,d\right){|{\Phi }_{0,0}|}^{2}|{\Phi }_{0,0}\rangle \equiv -{|{C}_{0,0}|}^{2}{\Theta }_{n,m}\left(d\right)$$

where

35 $${\Theta }_{n,m}\left(d\right)=\frac{1}{2{d}_{{\rm{eff}}}}\langle {\Phi }_{n,m}|S\left(z,d\right){|{\Phi }_{0,0}|}^{2}|{\Phi }_{0,0}\rangle$$

Numerically, Θ_*n*,*m*_(*d*) can be calculated by

36 $${\Theta }_{n,m}\left(d\right)=\frac{1}{2{d}_{{\rm{eff}}}}\frac{\pi }{L}{\int }_{-d}^{d}\left({\int }_{0}^{+{\infty }}{\phi }_{n}^{* }\left(r,z\right){\left|{\phi }_{0}\left(r,z\right)\right|}^{2}{\phi }_{0}\left(r,z\right){e}^{i2n\left[\frac{1}{2}{\xi }_{0}\left(z\right)-\frac{{\xi }_{0}\left(L\right)}{2L}z\right]+{im}\Omega z}{rdr}\right){dz}$$

where ${\phi }_{n}\left(r,z\right)=\frac{\sqrt{2/\pi }}{w\left(z\right)}{L}_{n}\left[2\frac{{r}^{2}}{{w}^{2}\left(z\right)}\right]{e}^{-\frac{{r}^{2}}{{w}^{2}\left(z\right)}}{e}^{-{ik}\frac{{r}^{2}}{2R\left(z\right)}}$. Equation (35) is an alias for Eq. (7) in the main text.

### Cavity degeneracy and density of states

In the Floquet framework, the normalized DOS is defined as^70^:

37 $$D\left(\varepsilon \right)=\frac{1}{N}\mathop{\sum }\limits_{n,m}\delta \left(\varepsilon -{\varepsilon }_{n,m}\right)$$

where *N* is the total number of states. All distinct Floquet states can be indexed by eigenvalues within the first Floquet Brillouin zone (FBZ) [${\varepsilon }_{0},{\varepsilon }_{0}+\Omega$]. For a degenerate cavity, the DOS peaks at the degenerate eigenvalues, whereas for a non-degenerate cavity, it approaches zero as $N\to {\infty }$. Thus, for a cavity characterized by degenerate indices (*u*, *v*), the DOS can be explicitly expressed as

38 $$D=\left\{\begin{array}{c}\frac{1}{u},{\rm{for\; a}}\left(u,v\right){\rm{degenerate\; cavity}}\\ 0,{\rm{for\; a\; non}}-{\rm{degenerate\; cavity}}\end{array}\right.$$

### Determination of the MCS condition

The cavity degeneracy and MCS conditions are governed by Eqs. (4) and (8) in the main text, respectively. For an MPC characterized by degeneracy indices (*u*, *v*), Eq. (4) relates the mirror focal length *F* and the effective cavity length *L*_eff_ as

39 $$\frac{1}{\sqrt{2F/{L}_{{\rm{eff}}}-1}}=\tan \left(\frac{v\pi }{2u}\right)$$

Using Eq. (8), the MCS condition further establishes a relation between the MCS medium length *d*_MCS_ and *L*_eff_,

40 $$\frac{({d}_{{\rm{MCS}}}/{n}_{0})/{L}_{{\rm{eff}}}}{\sqrt{2F/{L}_{{\rm{eff}}}-1}}=\tan \left(\frac{k\pi }{2u}\right),{\rm{k}}=\mathrm{1,2},\ldots ,{\rm{v}}$$

Dividing Eq. (40) by (39) yields

41 $$\frac{{d}_{{\rm{MCS}}}}{{n}_{0}{L}_{{\rm{eff}}}}=\frac{\tan \left(k{\rm{\pi }}/2u\right)}{\tan \left(v{\rm{\pi }}/2u\right)}$$

which is equivalent to Eq. (9) of the main text.

Equations (39) and (41) provide a practical procedure for determining the MCS medium length. Specifically, for given indices (*u*, *v*) and *k*, together with a chosen cavity length 2 *L*, the MCS medium length *d*_MCS_ can be obtained self-consistently using ${L}_{{\rm{eff}}}=\left(L-{d}_{{\rm{MCS}}}\right)+\frac{{d}_{{\rm{MCS}}}}{{n}_{0}}$. Once *L*_eff_ is determined, the required mirror focal length *F* follows directly from Eq. (39).

Alternatively, if the mirror focal length *F* is specified a priori, Eq. (39) can first be used to determine *L*_eff_ for a given degeneracy (*u*, *v*), after which the corresponding MCS medium length *d*_MCS_ can be obtained from Eq. (41).

### Critical power constraint

The critical power for self-focusing in a Kerr medium is approximately^75^

42 $${P}_{{\rm{cr}}}\approx {\lambda }_{0}^{2}/\left(2{\rm{\pi }}{n}_{0}{n}_{2}\right)$$

According to Eq. (24), the incident peak power is related to SNLP as

43 $${P}_{0}=\frac{{\rm{\pi }}{w}_{0}^{2}{\lambda }_{0}b}{8{\rm{\pi }}{n}_{2}{d}_{{\rm{eff}}}}$$

where *w*_0_ is the beam waist.

For gas-filled MPCs, imposing the critical-power constraint ${P}_{0}/{P}_{{\rm{cr}}} < 1$ yields

44 $$b < 4\arctan \left(\frac{d}{{z}_{0,{\rm{Kerr}}}}\right)$$

where ${z}_{0,{\rm{Kerr}}}={n}_{0}{L}_{{\rm{eff}}}\sqrt{\frac{2F}{{L}_{{\rm{eff}}}}-1}$ is the effective Rayleigh length. We also consider the relaxed constraint, where ${P}_{0}/{P}_{{\rm{cr}}} > 1$ is allowed, while the self-focusing point remains outside the Kerr medium (*z*_SF_ > 2 *d*). The self-focusing length can be empirically estimated as^72^

45 $${z}_{{\rm{SF}}}=\frac{0.367{z}_{0,{\rm{Kerr}}}}{\sqrt{{\left[{\left({P}_{0}/{P}_{{\rm{cr}}}\right)}^{\frac{1}{2}}-0.852\right]}^{2}-0.0219}}$$

which yields

46 $${b < 4\arctan \left(\frac{d}{{z}_{0,{\rm{Kerr}}}}\right)\left[\sqrt{{\left(\frac{0.184}{d/{z}_{0,{\rm{Kerr}}}}\right)}^{2}+0.0219}+0.852\right]}^{2}$$

Equations (44) and (46) are equivalent to Eqs. (10) and (11) of the main text, respectively. In Supplementary Fig. S4, we plot *b*_max_ as a function of *d*/*z*_0,Kerr_ obtained from Eqs. (10) and (11).

## Supplementary information

Supplementary Materials

## Data Availability

The data that support the plots in the main text of this paper are openly available via Zenodo at 10.5281/zenodo.20064654. Additional data can be obtained upon request from the corresponding author, Z. T.
