# Supplementary material for: Stabilization and destabilization of multimode solitons in nonlinear degenerate multi-pass cavities
Source: Light Sci Appl. 2026 Jun 26;15:287. doi: 10.1038/s41377-026-02327-0 (PMC13309540; doi:10.1038/s41377-026-02327-0)
Supplement: Supplementary file 1 — Supplementary Materials [file 41377_2026_2327_MOESM1_ESM.docx]

**Supplementary Materials for**

**Stabilization and Destabilization of Multimode Solitons in Nonlinear Degenerate Multi-Pass Cavities**

Junhan Huang^1,2^, Bingbing Zhu^1,2^, Shanyue Li^1,2^, Kun Ding^1,2^, and Zhensheng Tao^1,2*^

^1^ State Key Laboratory of Surface Physics, Key Laboratory of Micro and Nano Photonic Structures (MOE), and Department of Physics, Fudan University, Shanghai 200433, China.

^2^ Shanghai Key Laboratory of Metasurfaces for Light Manipulation, Fudan University, Shanghai 200433, China.

*Corresponding author. Email: [zhenshengtao@fudan.edu.cn](mailto:zhenshengtao@fudan.edu.cn).

**Table of Contents**

**__________________________________________________________**

Section S1. [Details and Benchmark of NLSE Simulations](#_Toc375303395) 3

Section S2. [Propagation of Stable Spatial Discrete Solitons](#_Toc375303395) 5

Section S3. Fox-Li Algorithm for Soliton Solutions 6

Section S4. Soliton Stability Analysis 7

Section S5. Derivation of Overlap Integral and MCS Length 7

Section S6. [Phase Diagrams Calculated Using Floquet Analysis](#_Toc375303406) 8

Section S7. [Critical Power Constraint](#_Toc375303406) 9

Section S8[. Robustness of MCS conditions](#_Toc375303406) 10

Section S9. Thermal Lensing Effect 16

Section S10. Supplementary Data 19

References 19

**S1. Details and Benchmark of NLSE Simulations**

For the NLSE simulations, the temporal grid is uniform with 256 points over a −1 to 1 ps window. The radial grid is non-uniform, defined by$r=r_{0}(e^{y}-1)$ with *y* uniformly discretized and *r*_0_=200 μm; the full grid extends to *r*_max_=3000 μm with 1024 radial points. The axial propagation step is also non-uniform: inside the Kerr medium, we use a step size of *d*/1500, where 2*d* is the medium thickness; in vacuum, the step size is (*L*−*d*)/750, where 2*L* is the cavity length.

The accuracy of our NLSE simulations was benchmarked against the spectral broadening and transform-limited pulse durations reported in previous experimental studies.

For the solid MPC, we conducted simulations using the parameters reported in Ref. 1: *F*=300 mm, 2*L*=520 mm, and a fused-silica medium with 2*d*=9.5 mm. The input pulses had a duration of 470 fs and an energy of 20.5 μJ. The simulated broadened spectra after 31 passes (Figs. S1a-b) yield a transform-limited duration of 64 fs, in close agreement with the experimental result of 72 fs and the reported transform-limited duration of 68 fs. The spectral bandwidth obtained from our simulation also matches well with the measured data.

For the gas-filled MPC, we followed the configuration of Ref. 2, using *F*=150 mm, 2*L*=286.5 mm, and 7 bar of argon (*n*_2_=6.5⨉10^-22^ m^2^ W^-1^ and *k’’*=0.110 fs^2^ mm^-1^) as the nonlinear medium. The input pulses had a duration of 275 fs and an energy of 160 μJ. The simulated spectra (Figs. S1c-d) yield a transform-limited duration of 31 fs, again in excellent agreement with the experimental result of 33 fs. The simulated spectral broadening reproduces both the bandwidth and shape observed in the experiment.

These benchmarks confirm that our NLSE framework reliably captures the key features of spectral broadening and pulse compression in both solid and gas-filled MPCs.


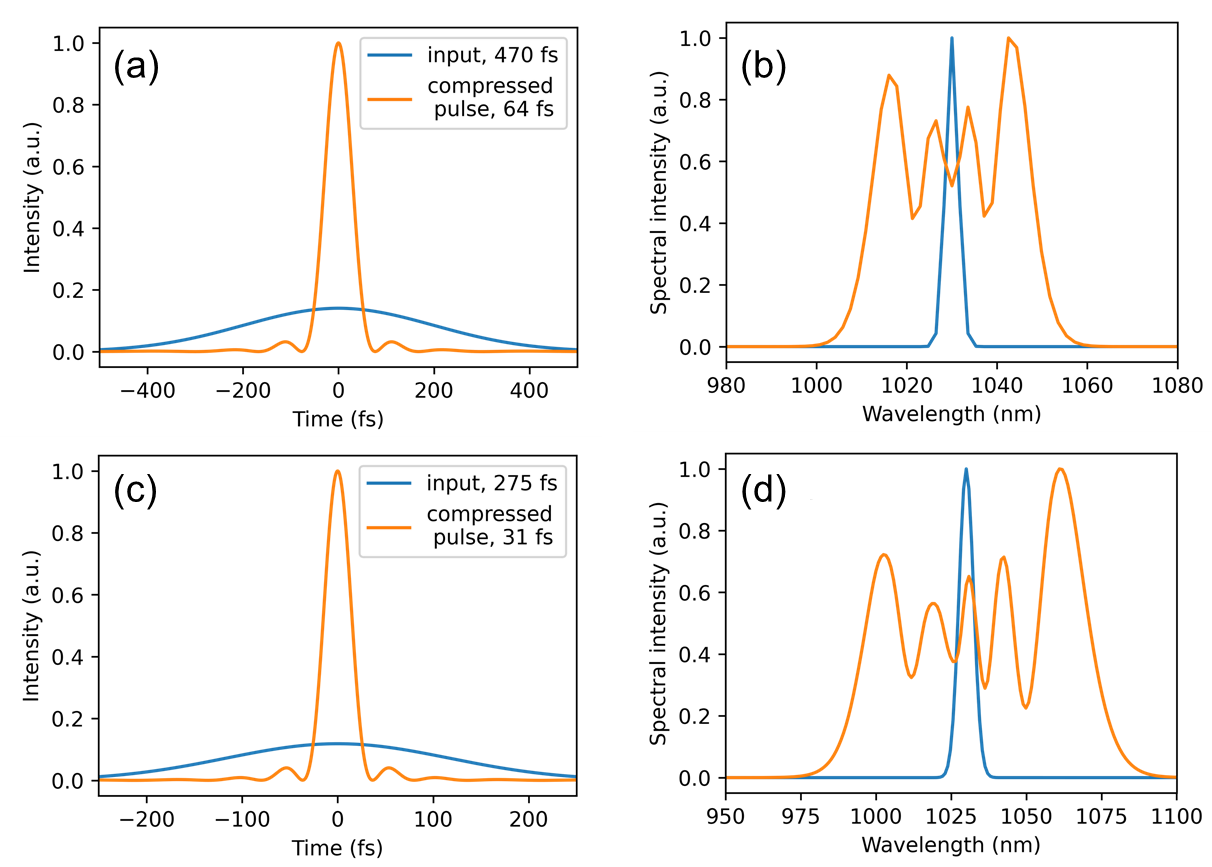


**Figure S1. (a-b)** Transform-limited pulse durations and spectra of the input and compressed pulses obtained from NLSE simulations using parameters in Ref. 1, in direct comparison with Figs. 4c and d in Ref. 1. **(c-d)** Same as **(a-b)**, but for the results in Figs. 2a and b in Ref. 2.

**S2.** **Propagation of Stable Spatial Discrete Solitons**

In Fig. S2, we plot the beam propagation dynamics for conditions A and B as shown in Fig. 2 of the main text. It is clear that for condition B, the laser pulses maintain stable spatial profiles within the nonlinear medium and on the cavity mirrors, indicating the formation of stable spatial discrete solitons. However, for condition A (degenerate cavity geometry), the spatial profile distorts as the beam propagates forward, and the temporal profile undergoes strong splitting.


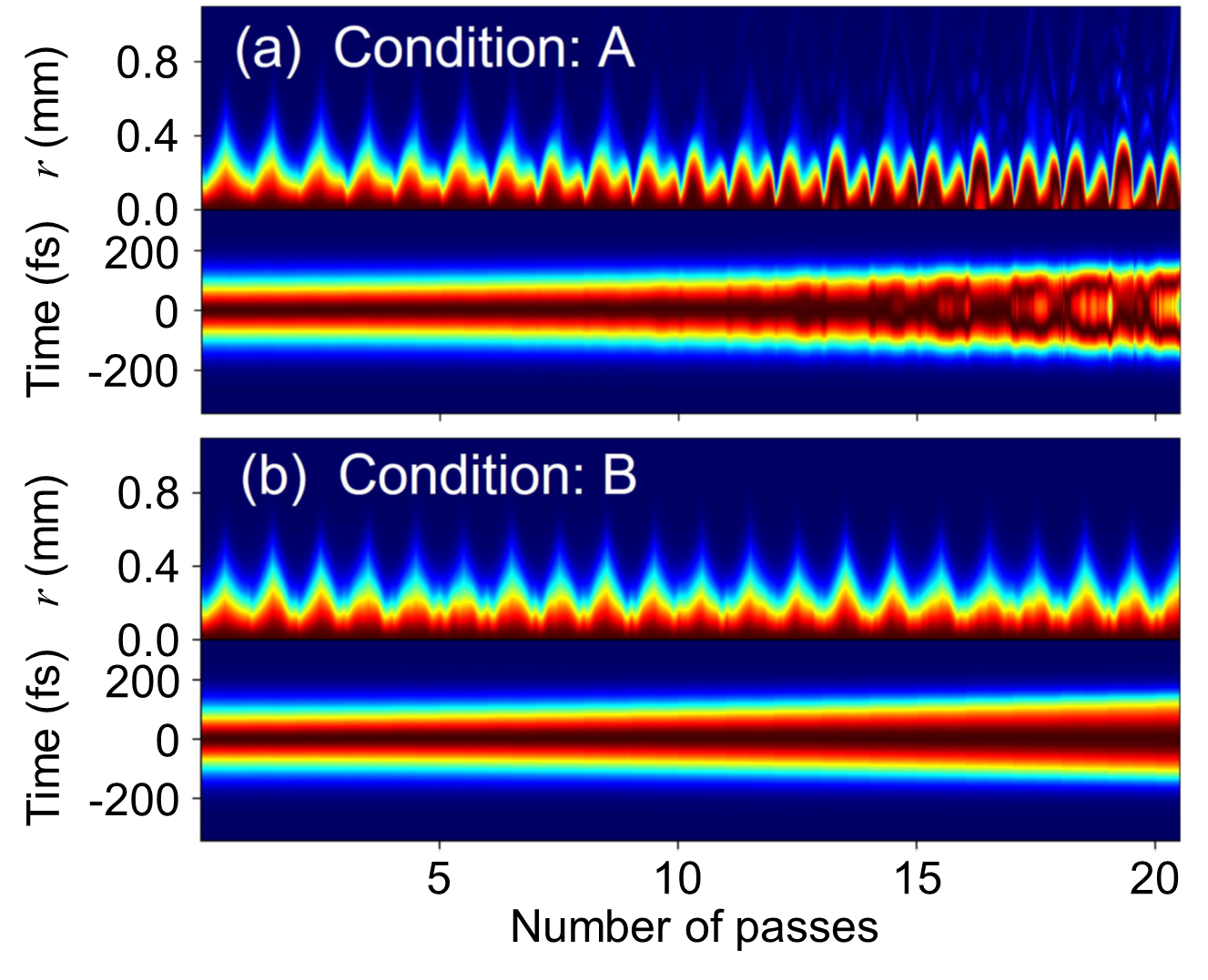


**Figure S2.** Beam propagation dynamics corresponding to conditions A and B. A unit linear refractive index is assumed (*n*_0_=1). Material dispersion is neglected.

**S3. Fox-Li Algorithm for Soliton Solutions**

For a solid MPC, under thin-lens and thin-Kerr-medium approximations, the simplified NLSE has an equivalent integral form, where the propagation in free space is described by Fresnel-Kirchhoff diffraction (FKD), and the effects of the lens and the Kerr medium is represented by the phase factors $e^{i\frac{\pi r^{2}}{\lambda_{0}F}}$ and $e^{ib\left| U \right|^{2}}$. The FKD integral for free-space propagation with length *L* (half the cavity) is given by

$\hat{\mathcal{F}}U\left( \rho\right)=-2\pi ie^{i\pi\rho^{2}}\int_{0}^{+\infty} U\left( \rho' \right)e^{i\pi{\rho'}^{2}}J_{0}\left( 2\pi\rho\rho' \right)\rho'd\rho'$, (S1)

where *J*_0_ is the zeroth-order Bessel function, and $\rho=r/{\sqrt{\lambda_{0}L}}$ is the reduced radial coordinate. The propagation from the *k*-th Kerr plate to the (*k*+1)-th plate is given by

$\left. \left| U_{k+1}\left( \rho\right) \right. \right\rangle=\hat{\mathcal{L}}e^{ib\left| U_{k}\left( \rho\right) \right|^{2}}\left. \left| U_{k}\left( \rho\right) \right. \right\rangle$. (S2)

Here, $\hat{\mathcal{L}}\equiv\hat{\mathcal{F}}\hat{\mathcal{N}}\hat{\mathcal{F}}$ describes the linear part of beam propagation, where $\hat{\mathcal{N}}\equiv e^{i\frac{\pi\rho^{2}}{F/L}}$ represents the focus of the spherical mirrors, and $e^{ib\left| U\left( \rho\right) \right|^{2}}$ represents the nonlinear self-focusing induced by a thin Kerr plate.

This model can be solved following the Fox-Li iteration framework^3,4^. The idea here is to find the soliton solution that satisfies $\left| {U_{k+1}\left( \rho\right)}/{U_{k}\left( \rho\right)} \right|\to1$ using the Fox-Li algorithm. The Fox-Li pseudocode is as follows:


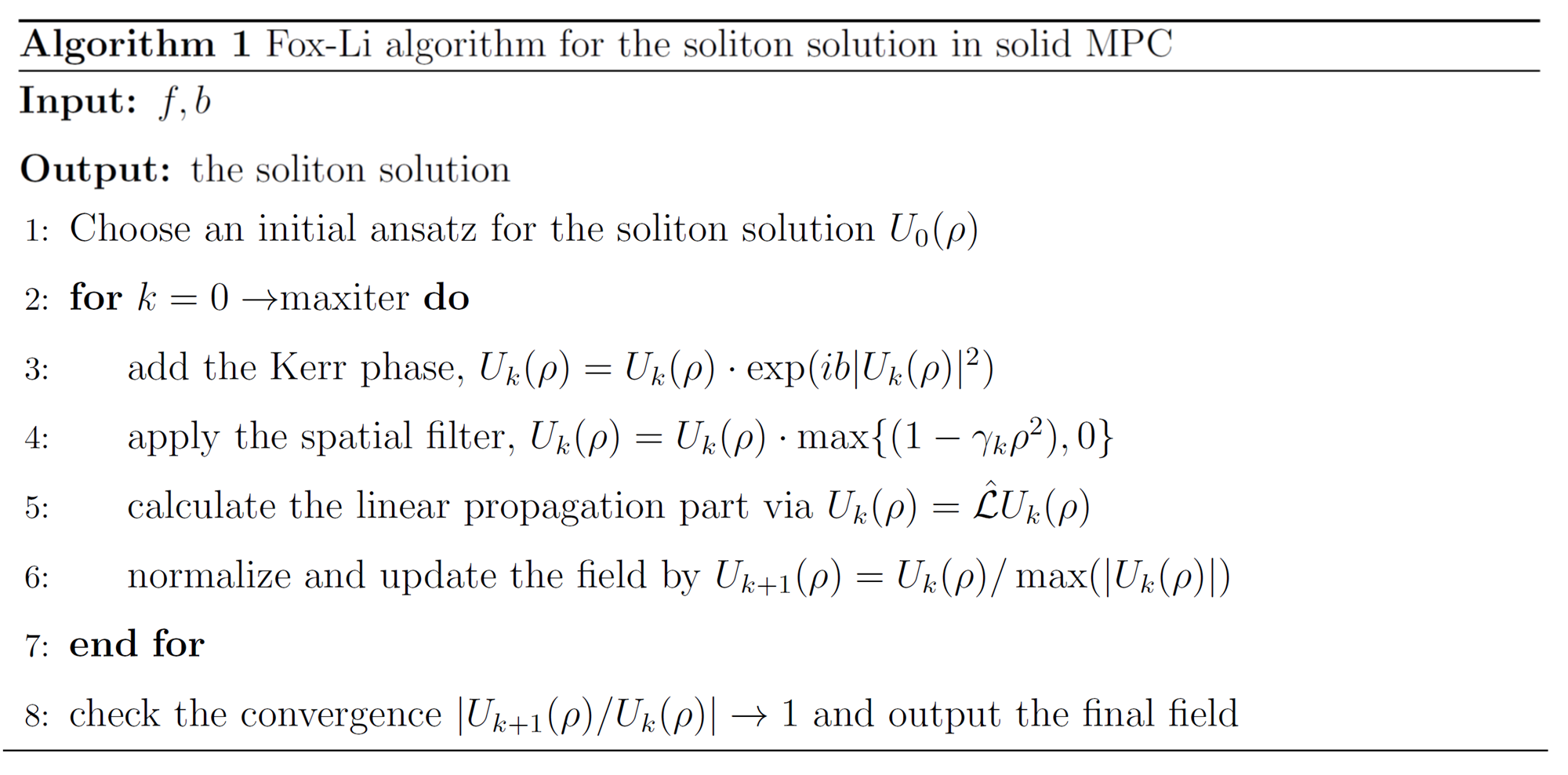


For the Fox–Li iteration algorithm, we employ a uniform radial grid in the normalized coordinate $\rho=r/\sqrt{\lambda L}$ with 16000 points spanning 𝜌=0 to 5. A typical calculation uses 2000 iterations, with convergence determined when the relative change of the field between successive iterations falls below 10^-5^.

**S4. Soliton Stability Analysis**

To analyze the stability of the soliton solutions, we employ a linear stability analysis approach^5^. We perform the standard linearization procedure, introduce a small perturbation to the soliton solution, and use the perturbed solution as the initial condition in the simplified NLSE simulations. Specifically, we choose the perturbation to be $\left. \left| w \right. \right\rangle=\sum_{n} a_{n}\left. \left| \psi_{n} \right. \right\rangle$, where $\left. \left| \psi_{n} \right. \right\rangle$ is the LG*_n_* mode and *a_n_* is a random coefficient in the interval [0.02, 0.04] ^6^.

**S5. Derivation of Overlap Integral and MCS Length**

The overlap integral has been derived from the perturbative analysis, which is given by

$\Theta_{n,m}\left( d \right)=\frac{1}{2d_{\mathrm{eff}}}\left\langle\Phi_{n,m}\left( r,z \right) | S\left( z, d \right)\left| \Phi_{0,0}\left( r,z \right) \right|^{2} | \Phi_{0,0}\left( r,z \right) \right\rangle$. (S3)

For the convenience of calculation, we introduce

$\phi_{n}\left( r,z \right)=\frac{\sqrt{2/\pi}}{w\left( z \right)}L_{n}\left[ 2\frac{r^{2}}{w^{2}\left( z \right)} \right]e^{-\frac{r^{2}}{w^{2}\left( z \right)}}e^{-ik\frac{r^{2}}{2R\left( z \right)}}$, (S4)

which yields the Floquet state

$\left. \left| \Phi_{n,m}\left( r,z \right) \right. \right\rangle=\phi_{n}\left( r,z \right)e^{-i\frac{1}{2}\xi_{n}\left( z \right)+i\frac{\xi_{n}\left( L \right)}{2L}z-im\Omega z}$. (S5)

Substituting Eq. (S5) into Eq. (S3), we obtain

$\Theta_{n,m}\left( d \right)=\frac{1}{2d_{\mathrm{eff}}}\frac{\pi}{L}\int_{-d}^{d} \left( \int_{0}^{+\infty} \phi_{n}^{*}\left( r,z \right)\left| \phi_{0}\left( r,z \right) \right|^{2}\phi_{0}\left( r,z \right)e^{i2n\left[ \frac{1}{2}\xi_{0}\left( z \right)-\frac{\xi_{0}\left( L \right)}{2L}z \right]+im\Omega z}rdr \right)dz$. (S6)

The integral with respect to *r* can be analytically calculated as

$$\int_{0}^{+\infty} \phi_{n}^{*}\left( r,z \right)\left| \phi_{0}\left( r,z \right) \right|^{2}\phi_{0}\left( r,z \right)rdr=\int_{0}^{+\infty} \frac{1}{{w\left( z \right)}^{4}}\left( \frac{2}{\pi} \right)^{2}L_{n}\left( 2\frac{r^{2}}{{w\left( z \right)}^{2}} \right)e^{-4\frac{r^{2}}{{w\left( z \right)}^{2}}}rdr$$

$\mathbf{=}\frac{1}{{w\left( z \right)}^{2}}\frac{1}{\pi^{2}}\int_{0}^{+\infty} L_{n}\left( x \right)e^{-2x}dx=\frac{1}{{w\left( z \right)}^{2}}\frac{1}{\pi^{2}}\frac{1}{2^{n+1}}$ (S7)

with the substitution of variable $2\frac{r^{2}}{{w\left( z \right)}^{2}}\to x$.

The integral with respect to *z* can be evaluated analytically in the case when the cavity is degenerate, i.e., $\varepsilon_{n,m}=\varepsilon_{0,0}$ or equivalently $\frac{\xi_{n}\left( L \right)-\xi_{0}\left( L \right)}{2L}=\frac{2n\xi_{0}\left( L \right)}{2L}=m\Omega$. Thus, the overlap integral can be simplified to

$\Theta_{n,m}\left( d \right)=\frac{1}{\pi d_{\mathrm{eff}}L}\frac{1}{2^{n+2}}\int_{-d}^{d} \frac{1}{{w\left( z \right)}^{2}}e^{+i2n\frac{1}{2}\xi_{0}\left( z \right)}dz$

$=\frac{1}{\pi d_{\mathrm{eff}}}\frac{1}{L}\frac{1}{2^{n+2}}\frac{z_{0,\mathrm{Kerr}}}{w_{0}^{2}}\int_{-d}^{d} \frac{1}{1+\left( \frac{z}{z_{0,\mathrm{Kerr}}} \right)^{2}}e^{+i2n\arctan\left( \frac{z}{z_{0,\mathrm{Kerr}}} \right)}d\left( \frac{z}{z_{0,\mathrm{Kerr}}} \right)$ $=\frac{1}{2^{n+2}d_{\mathrm{eff}}}\frac{1}{L}\frac{z_{0,\mathrm{Kerr}}}{\pi w_{0}^{2}}\frac{\sin\left[ 2n\arctan\left( \frac{d}{z_{0,\mathrm{Kerr}}} \right) \right]}{n}$ $=\frac{n_{0}}{\lambda_{0}L}\frac{1}{n2^{n+2}}\frac{\sin\left[ 2n\arctan\left( \frac{d}{z_{0,\mathrm{Kerr}}} \right) \right]}{z_{0,\mathrm{Kerr}}\arctan\left( \frac{d}{z_{0,\mathrm{Kerr}}} \right)}$, (S8)

where $d_{\mathrm{eff}}=z_{0,\mathrm{Kerr}}\arctan\left( \frac{d}{z_{0,\mathrm{Kerr}}} \right)$ represents an effective medium length, and $z_{0,\mathrm{Kerr}}=n_{0}L_{\mathrm{eff}}\sqrt{\frac{2F}{L_{\mathrm{eff}}}-1}$ is the effective Rayleigh length, with $L_{\mathrm{eff}}=\left( L-d \right)+\frac{d}{n_{0}}$ being the effective cavity length. By setting the overlap integral in Eq. (S8) equal to zero, we obtain

$4u\arctan\left( \frac{d_{\mathrm{MCS}}}{z_{0,\mathrm{Kerr}}} \right)=4u\arctan\left( \frac{d_{\mathrm{MCS}}/L_{\mathrm{eff}}}{n_{0}\sqrt{2F/L_{\mathrm{eff}}-1}} \right)=2k\pi, k=1, 2, \cdots,v$ (S9)

**S6.** **Phase Diagrams Calculated Using Floquet Analysis**

In the main text, we analyze the stability conditions of multimode solitons within the Floquet framework and derived soliton solutions using perturbation theory in the small-*b* limit. To test the validity of neglecting space-time coupling in this regime, we calculated soliton stability phase diagrams for nonlinear MPCs using Floquet analysis (Figs. S3a-b), and compared them with the phase diagrams obtained from the NLSE simulations (Figs. 2a-b).

For this purpose, we introduce the parameter $\chi$, which quantifies the higher-order contributions to the soliton solution, given by $\chi=\frac{\sum_{n\neq0} \left| C_{n} \right|^{2}}{\sum_{n} \left| C_{n} \right|^{2}}$.

**
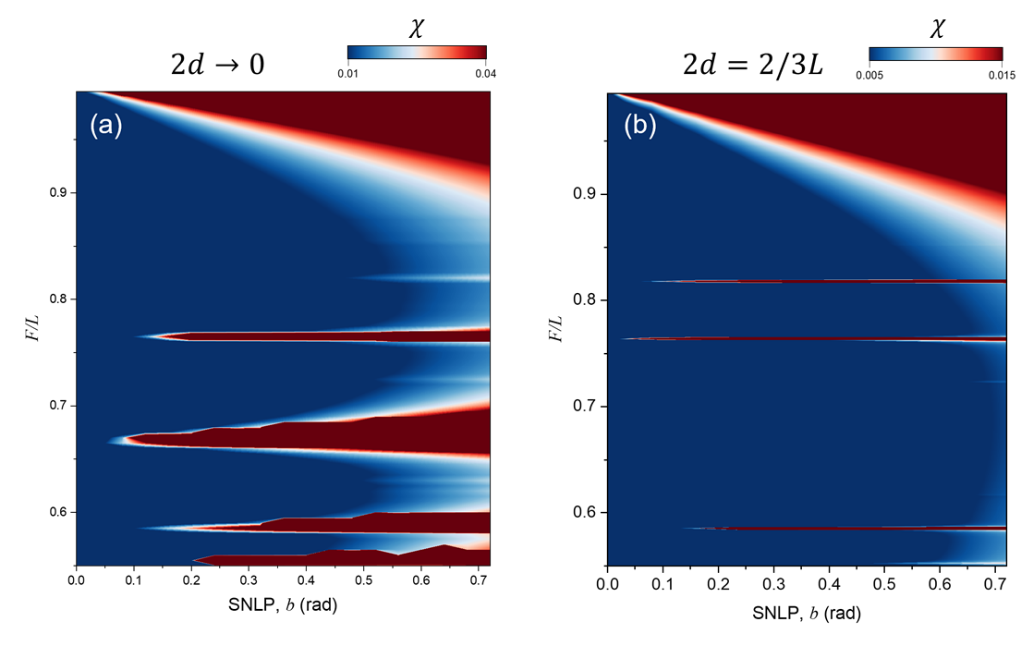
**

**Figure S3.** **(a-b)** Phase diagrams of higher-order mode contributions as a function of cavity geometry *F*/*L* and SNLP *b*, for medium length *d*/*L* corresponding to **(a)** a thin plate ($d\to0$), **(b)** the MCS length (*d*_MCS_). These results are in direct comparison with Figs. 2a-b in the main text. A unit linear refractive index is assumed (*n*_0_=1). Material dispersion is neglected.

**S7. Critical Power Constraint**


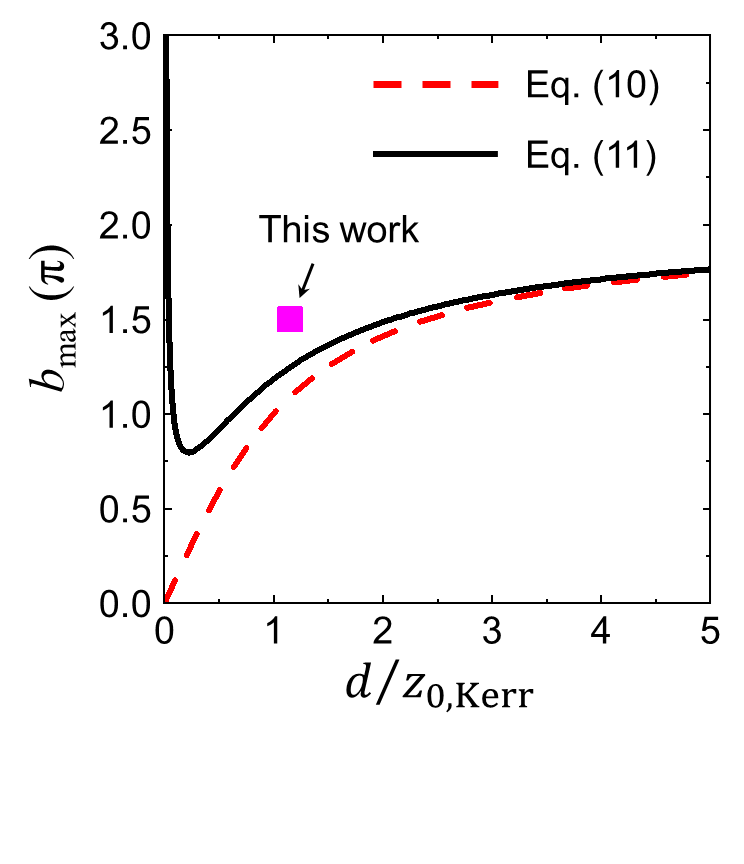


**Figure S4.** Maximum SNLP (*b*_max_) as a function of *d*/*z*_0,Kerr_ obtained from Eq. (10) and Eq. (11) of the main text.

**S8.** **Robustness of MCS conditions**

In this section, we discuss the practical feasibility of implementing the MCS condition. To examine its sensitivity to real-world variables, such as manufacturing tolerances, alignment inaccuracy, and uncertainties in material parameters, we systematically examined a range of practical perturbations based on the simulations in Figs. 5a-b. These perturbations include cavity mirror losses, degraded incident beam quality, beam divergence effects, longitudinal offsets of the Kerr medium, and cavity mirror dispersion.

1. **Mirror loss**

In Fig. S5a, we summarize the spatio-spectral homogeneity $\left\langle V \right\rangle$ and the transform-limited (TL) pulse durations as a function of mirror loss, assuming each cavity mirror has a loss up to 3%. A mirror loss of 3% already corresponds to relatively poor coating quality in practice. Our results show that the stability of the MCS condition is unaffected by mirror loss (Figs. S5b-c): loss reduces the circulating pulse energy on each pass, thereby decreasing the SNLP parameter *b* and pushing the system further into the stable regime. The primary consequence is reduced nonlinear accumulation. For example, after 18 passes, the achievable pulse compression is limited from 12.5 fs (0% loss) to 14 fs (3% loss).

Importantly, the high SNLP values enabled by the MCS condition requires only a small number of roundtrips, making the approach inherently resilient to loss. With a 1% mirror loss per pass, a conventional nonlinear MPC would typically incur 30%-50% total energy loss, whereas our MCS-assisted design results in only ~18% loss.


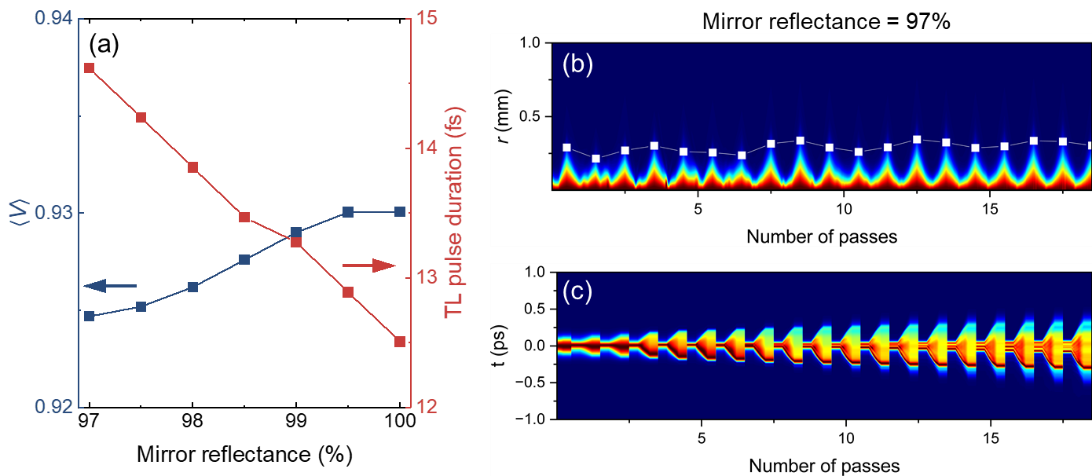


**Figure S5. (a)** Spatio-spectral homogeneity $\left\langle V \right\rangle$ and TL pulse duration as functions of cavity mirror reflectance. **(b)** Beam-profile evolution and **(c)** temporal pulse evolution obtained from NLSE simulations for femtosecond pulses with a cavity mirror loss of 3%. Simulations are performed under the same conditions as in Fig. 5a-b of the main text.

1. **Spatial Mode Variations**

We perform NLSE simulations for incident beams with beam-quality factor M^2^ ranging from 1.0 to 1.8. The incident beam is modeled as a super-Gaussian profile, $U\left( r \right)=exp\left( -\left( \frac{r}{w_{0}} \right)^{p} \right)$, with the corresponding M^2^ determined by $M^{2}=\frac{p\sqrt{\Gamma\left( \frac{3}{p} \right)\Gamma\left( 2-\frac{1}{p} \right)}}{\Gamma\left( \frac{1}{p} \right)}$. The incident pulse energy is slightly adjusted in each case to ensure the same SNLP parameter *b* as in Figs. 5a-b.

In Fig. S6a, we summarize $\left\langle V \right\rangle$and the TL pulse durations of the output beam after 18 passes. The results show that spatial-mode degradation of the incident beam does not compromise the beam-propagation stability ensured by the MCS condition. Typical propagation dynamics and pulse evolution for M^2^=1.6 are shown in Fig. S6b-d. In Fig. S6e, we further present the modal decomposition of the output beam for different M^2^ values. Notably, no selective enhancement of higher-order modes is observed in all the cases, indicating that multimode coupling remains effectively suppressed even when the incident beam quality is significantly degraded. The main effect of increasing M^2^ is a moderate reduction of the spatio-spectral homogeneity from 0.93 to 0.88 as M^2^ increases from 1.0 to 1.8.

Such robustness can be understood from the underlying MCS mechanism. An incident beam with arbitrary M^2^ can be decomposed into a superposition of LG modes. Under the MCS condition, nonlinear coupling between different LG modes is suppressed, such that each mode propagates quasi-independently through the nonlinear medium. The output beam therefore is composed of independently spectrally broadened modes, and the reduction of $\left\langle V \right\rangle$ arises from the distinct spatial dependence of nonlinear spectral broadening for higher-order LG modes.


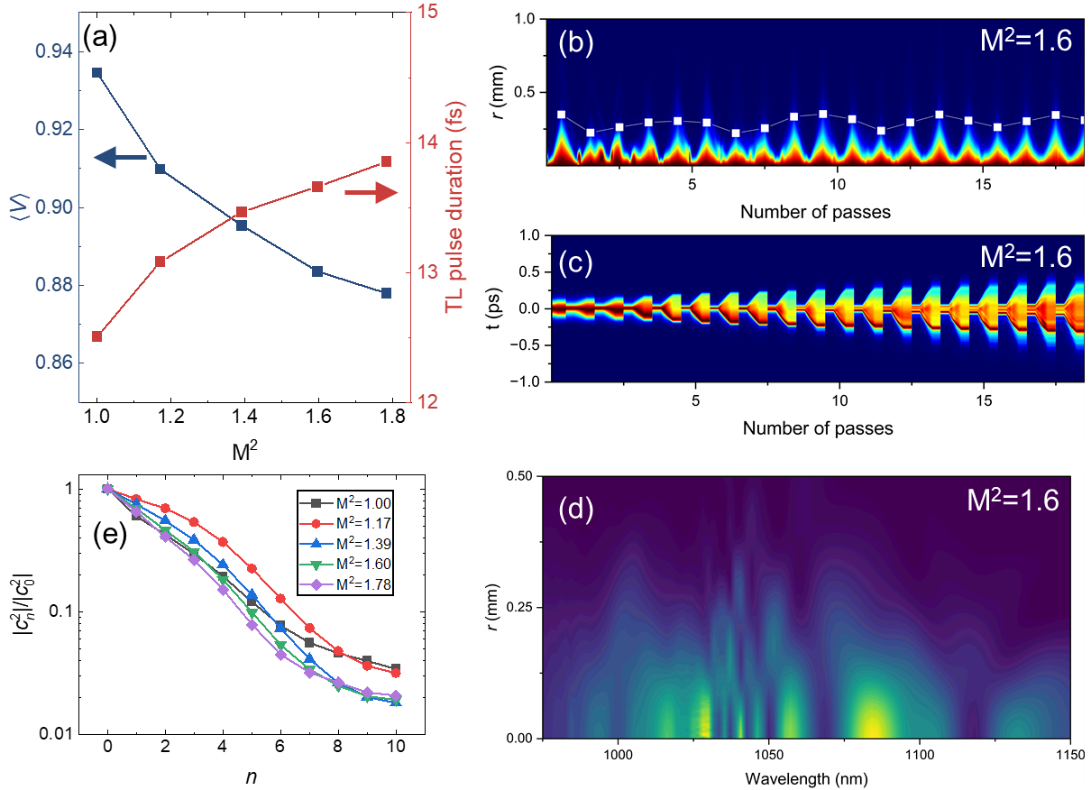


**Figure S6. (a)** Spatio-spectral homogeneity $\left\langle V \right\rangle$ and TL pulse duration as functions of incident beam M^2^. **(b)** Beam-profile evolution and **(c)** temporal pulse evolution obtained from NLSE simulations for femtosecond pulses with M^2^=1.6. **(d)** Radial distribution of the output pulse spectrum obtained from the simulations in **(b-c). (e)** Normalized expansion coefficients ${\left| c_{n} \right|^{2}}/{\left| c_{0} \right|^{2}}$ of the LG*_n_* modes for different M^2^ values.

1. **Beam Divergence Effect**

We examine the beam divergence effect by varying the incident beam waist as $w^{'}=\left( 1+\Delta\right)w_{0}$, where $w_{0}=\sqrt{\frac{\lambda_{0}L_{\mathrm{eff}}}{\pi}}\left( \frac{2F}{L_{\mathrm{eff}}}-1 \right)^{1/4}$ represents the eigenmode waist of the corresponding linear cavity. When the incident beam waist is varied from $\Delta=$-40% to 10%, stable propagation under the MCS condition is maintained (see Fig. S7a, and S7b-e). Increasing the incident beam waist leads to tighter focusing within the cavity, resulting in stronger nonlinearity and shorter transform-limited pulse durations. For Δ>10%, beam-propagation instabilities begin to emerge.


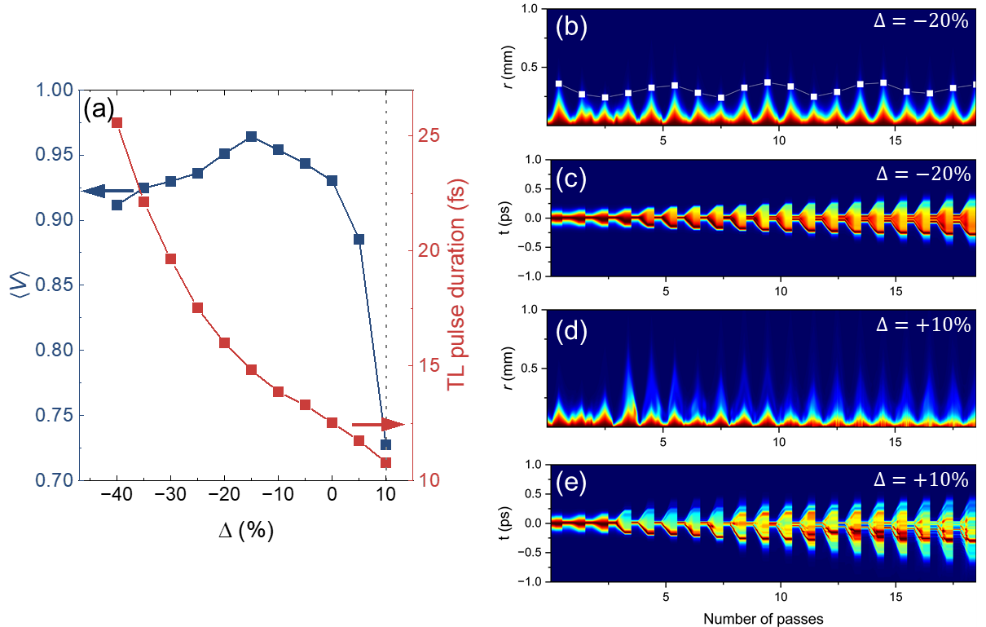


**Figure S7. (a)** Spatio-spectral homogeneity $\left\langle V \right\rangle$ and TL pulse duration as functions of incident beam waist variations Δ. **(b)** Beam-profile evolution and **(c)** temporal pulse evolution obtained from NLSE simulations for femtosecond pulses with Δ=-20%. **(d-e)** Same as **(b-c)**, but for Δ=10%.

1. **Kerr Medium Displacement and Length Error**

Figure. S8 presents NLSE simulations in which the Kerr medium is displaced from the cavity center by up to 2.0 cm (~8% of the cavity length). The results show that MCS-enabled stability is preserved over this range of misalignment. In a second test, the medium length is varied by up to ${\Delta d}/{d_{\mathrm{MCS}}}$=±8% around the MCS value to simulate fabrication errors (Fig. S9). The simulations again confirm that the MCS condition remains robust against such deviations.


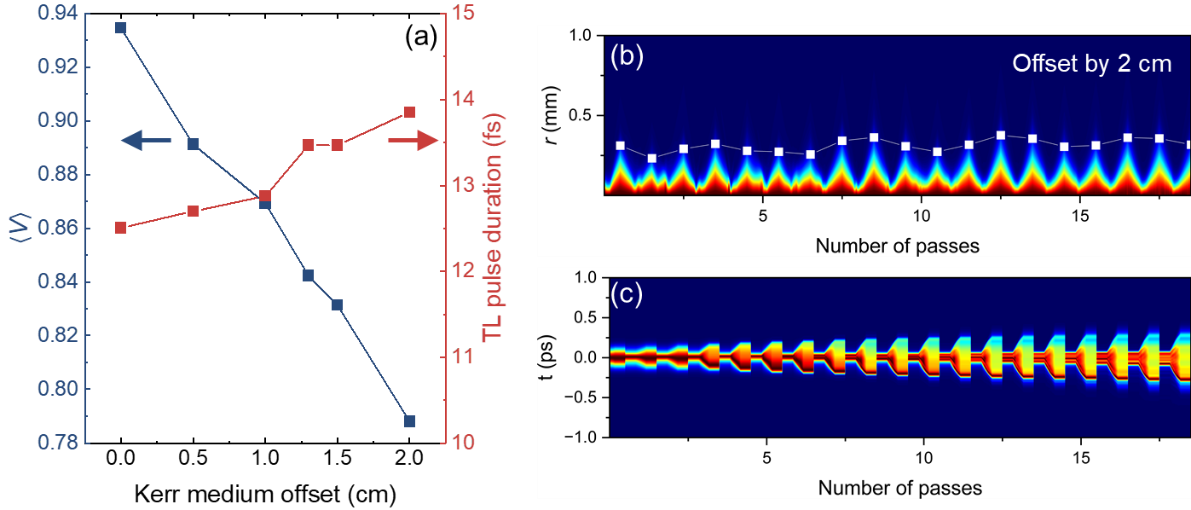


**Figure S8. (a)** Spatio-spectral homogeneity $\left\langle V \right\rangle$ and TL pulse duration as functions of Kerr-medium offset. **(b)** Beam-profile evolution and **(c)** temporal pulse evolution obtained from NLSE simulations for femtosecond pulses with the Kerr medium offset by 2 cm from the cavity center.


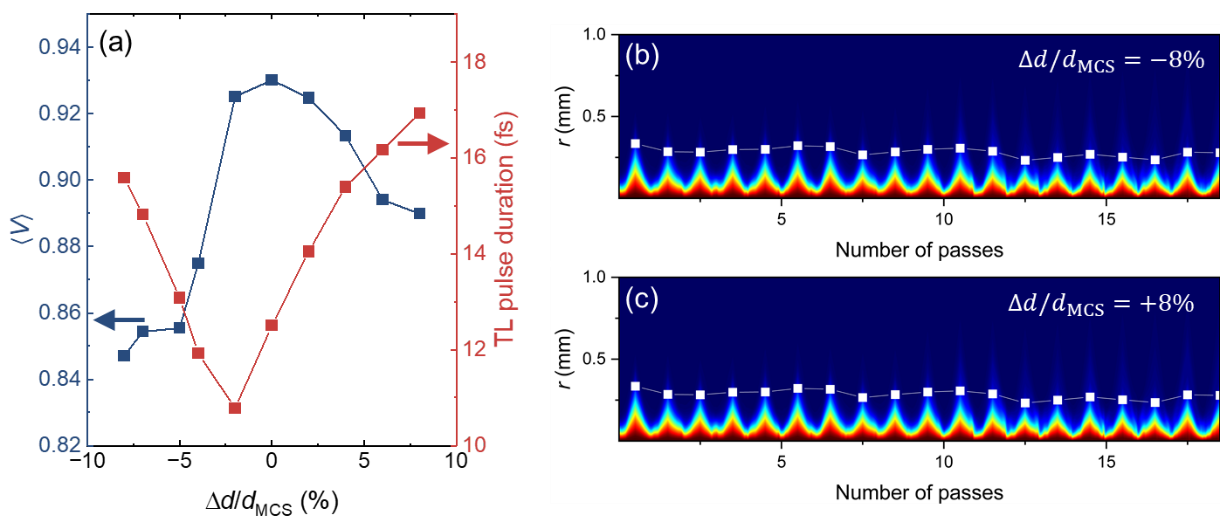


**Figure S9. (a)** Spatio-spectral homogeneity $\left\langle V \right\rangle$ and TL pulse duration as functions of the medium length perturbation (${\Delta d}/{d_{\mathrm{MCS}}}$). **(b)** Beam-profile evolution from NLSE simulations for femtosecond pulses with ${\Delta d}/{d_{\mathrm{MCS}}}$=-8%. **(c)** Same as **(b)**, but for ${\Delta d}/{d_{\mathrm{MCS}}}$=+8%.

1. **Kerr Medium Dispersion**

We first examine the role of GDD, i.e. second-order dispersion, of the Kerr medium. In the presence of space-time coupling, dispersion compensation is essential to maintain optimal nonlinear evolution in MPCs. In our simulations, the Kerr medium is fused silica with a total length of 10.67 cm, corresponding to a positive GDD. To compensate this dispersion, a negative GDD of approximately -1970 fs^2^ per cavity mirror is required.

Figure S10 summarizes the dispersion budget compatible with stable propagation under the MCS condition. When the negative dispersion provided by the mirrors is small (e.g., -1600 fs^2^; Figs. S10b–c), spatial stability is maintained. In this under-compensated regime, temporal pulse broadening reduces the peak intensity and hence the effective SNLP, pushing the system further into a stable operating region. As a result, the accumulated nonlinear phase is reduced, leading to longer transform-limited pulse durations (e.g., 48 fs at GDD=-400 fs^2^; see Fig. S10a).

In contrast, when the negative dispersion is increased beyond the optimal value (e.g., -2080 fs^2^; Figs. S10d–e), stable beam propagation breaks down. This is caused by the generation of excessively short pulses during the propagation, which significantly increases the SNLP and drives the system into an unstable regime. Our simulations show that for GDD<−2080 fs^2^, beam propagation becomes unstable even under the MCS condition.


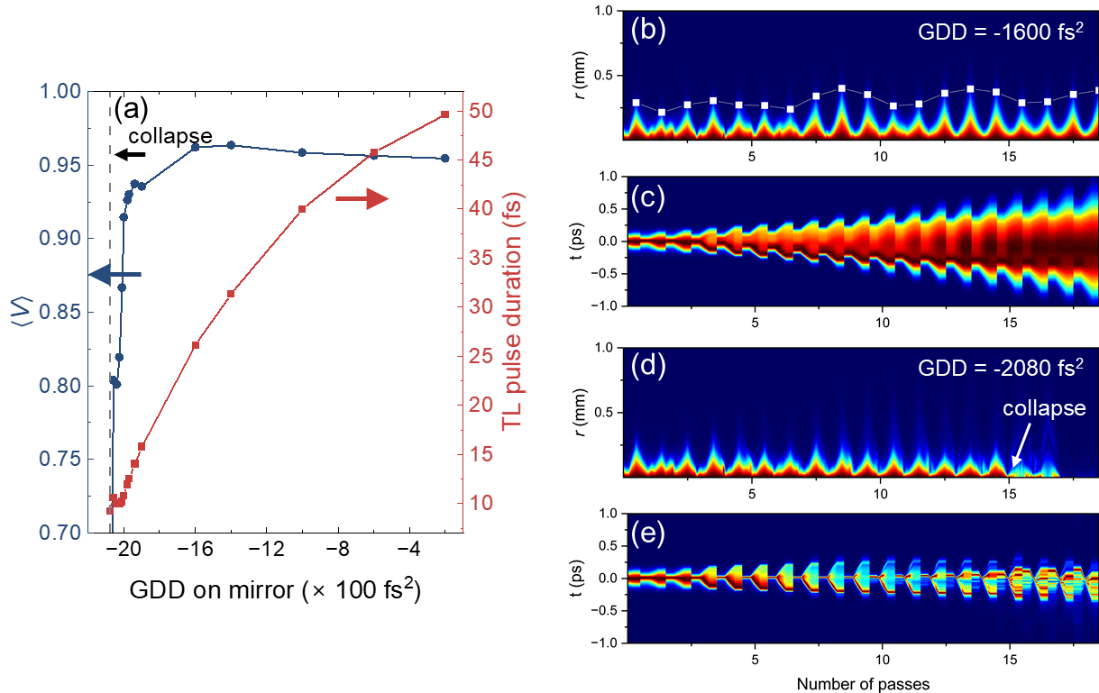


**Figure S10. (a)** Spatio-spectral homogeneity $\left\langle V \right\rangle$ and TL pulse duration as functions of mirror-compensated GDD. **(b)** Beam-profile evolution and **(c)** temporal pulse evolution obtained from NLSE simulations for femtosecond pulses for GDD=-1600 fs^2^. **(d-e)** Same as **(b-c)**, but for GDD=-2080 fs^2^. The position where beam collapse occurs is indicated.

In the simulations presented in Figs. 5a-b, higher-order dispersion of the Kerr medium is not considered. Here, we explicitly assess the influence of third-order dispersion (TOD) and its compensation by the cavity mirrors. For fused silica, the TOD per unit length is *k’’’*=41.15 fs^3^ mm^-1^, corresponding to a total TOD of ~4390 fs^3^ for a medium length of 10.67 cm. Figure S11a summarizes $\left\langle V \right\rangle$and the TL pulse durations of the output beams as functions of the mirror-provided TOD compensation. In all cases, stable beam propagation is maintained, and appropriate TOD compensation can slightly improve the spatio-spectral homogeneity $\left\langle V \right\rangle$. Representative spatial and temporal evolutions are shown in Figs. S11b-c for uncompensated TOD (TOD=0), and in Figs. S11d-e for optimal TOD compensation (TOD=-4390 fs^3^).


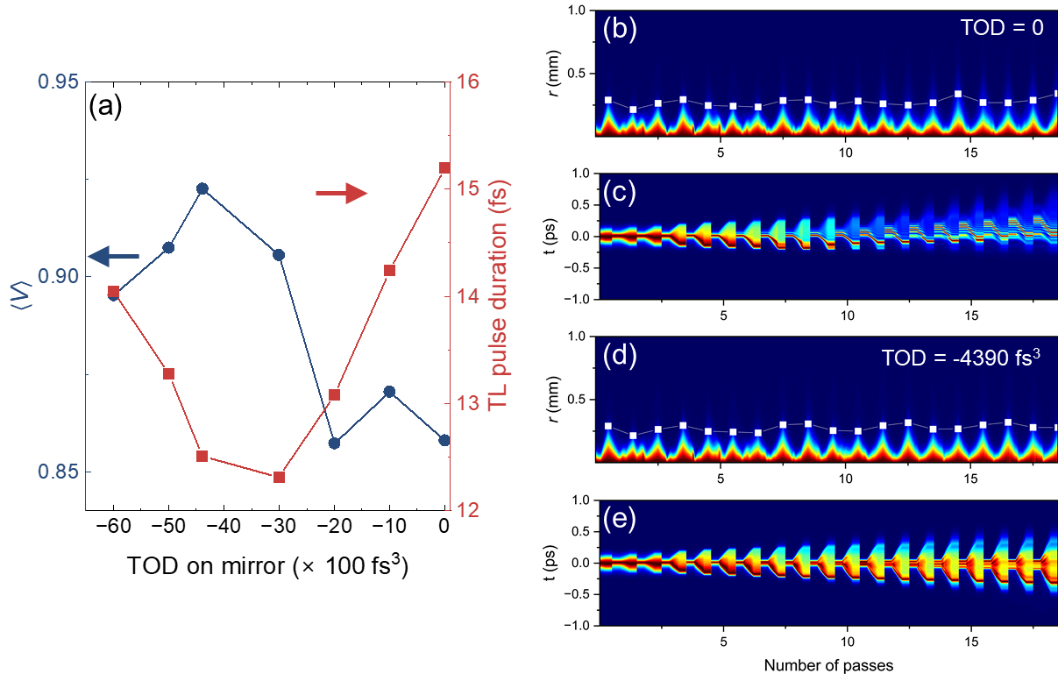


**Figure S11. (a)** Spatio-spectral homogeneity $\left\langle V \right\rangle$ and TL pulse duration as functions of mirror-compensated TOD. **(b)** Beam-profile evolution and **(c)** temporal pulse evolution obtained from NLSE simulations for femtosecond pulses for TOD=0. **(d-e)** Same as **(b-c)**, but for TOD=-4390 fs^3^.

**S9. Thermal Lensing Effect**

At high incident powers, thermal effects can be modeled as an additional thin lens in the Kerr medium or effectively modifying the focal length of the cavity mirrors. Here, we consider thermal lensing in the Kerr medium as a representative example. The focal lens of the thermal lens, *F*_therm_, can be estimated as

$F_{\mathrm{therm}}^{-1}=\frac{{\partial n}/{\partial T}}{2\kappa A}P_{\mathrm{heat}}$, (S10)

where ${\partial n}/{\partial T}$ and 𝜅 are the thermo-optic coefficient and thermal conductivity of the medium, respectively, *A* is the beam area, and *P*_heat_ is the dissipated power. Using typical values for fused silica (${\partial n}/{\partial T}$=5⨉10^-6^ K^-1^, 𝜅=1.35 W m^-1^K^-1^), a beam radius of 200 μm, and assuming 1% absorption of the incident power, we obtain $F_{\mathrm{therm}}\approx\frac{680 [\mathrm{cm}]}{P [W]}$. This yields *F*_therm_≈68 cm for *P*=10 W, and *F*_therm_≈6.8 cm for *P* = 100 W, indicating that thermal lensing starts to play a role at powers exceeding ~ 10 W.

We now discuss the implications for the MCS condition. The MCS regime is derived under the assumption of a *degenerate linear cavity*, which is also the configuration most commonly employed in practice to preserve the beam parameter *q* between the input and output of the cavity (the *q*-preserving condition). In a degenerate cavity characterized by indices (*u*, *v*), one observes *u* discrete laser spots on each cavity mirror (Fig. S12a). When thermal lensing is present, the cavity is detuned from degeneracy, and these discrete spots spread into continuous arcs (Fig. S12b).

This effect can be quantitatively modeled using the ABCD-matrix formalism by inserting an additional thin lens of focal length *F*_therm_ at the cavity center. Under the equivalent lens-sequence representation (Fig. S12c), the unit-cell ABCD matrix becomes

$\mathbf{M}=\left( \begin{matrix} 1 & 0 \\ -\frac{1}{F_{\mathrm{therm}}} & 1 \end{matrix} \right)\left( \begin{matrix} 1-\frac{L_{\mathrm{eff}}}{F} & n_{0}L_{\mathrm{eff}}\left( 2-\frac{L_{\mathrm{eff}}}{F} \right) \\ -\frac{1}{n_{0}F} & 1-\frac{L_{\mathrm{eff}}}{F} \end{matrix} \right)\equiv\left( \begin{matrix} A & B \\ C & D \end{matrix} \right)$. (S11)

The modified Gouy phase shift of the cavity is determined by the trace of **M**, specifically

$\xi'=2\arctan\sqrt{\frac{2F_{\mathrm{therm}}/\left( n_{0}L_{\mathrm{eff}} \right)+2F/L_{\mathrm{eff}}-1}{\left( 2F/L_{\mathrm{eff}}-1 \right)\left[ 2F_{\mathrm{therm}}/\left( n_{0}L_{\mathrm{eff}} \right)-1 \right]}}$. (S12)

Importantly, cavity degeneracy can be restored by adjusting the cavity length *L*🡪 *L’* ($L_{\mathrm{eff}}\to L_{\mathrm{eff}}^{'}=L^{'}-d+d/{n_{0}}$) such that $\xi'$ is restore to the Gouy phase shift before the presence of *F*_therm_, i.e.,

$\frac{2F_{\mathrm{therm}}/\left( n_{0}L_{\mathrm{eff}}^{'} \right)+2F/L_{\mathrm{eff}}^{'}-1}{\left( 2F/L_{\mathrm{eff}}^{'}-1 \right)\left[ 2F_{\mathrm{therm}}/\left( n_{0}L_{\mathrm{eff}}^{'} \right)-1 \right]}=\frac{1}{2F/L_{\mathrm{eff}}-1}$. (S13)

Solving this equation yields

$L_{\mathrm{eff}}^{'}=\left( F+\frac{F_{\mathrm{therm}}}{n_{0}} \right)-\sqrt{\left( F+\frac{F_{\mathrm{therm}}}{n_{0}} \right)^{2}-2L_{\mathrm{eff}}\frac{F_{\mathrm{therm}}}{n_{0}}}$. (S14)

As a concrete example, for the geometry used in Fig. 5 (*F*=5.88 cm, *L*=12.5 cm), assuming *F*_therm_=10 cm (corresponding to *P*~100 W), the cavity length should be adjusted to *L’*=11.97 cm to restore degeneracy. Experimentally, this condition can be readily identified by the reappearance of *u* discrete laser spots on the cavity mirrors.

In Fig. S13a, we present the performance of the MCS medium length under varying thermal-lensing strengths. In each case, the cavity degeneracy is restored by adjusting the cavity length, while all other parameters remain unchanged. For strong thermal lensing (*F*_therm_≈10 cm), stable nonlinear propagation can still be maintained over 9 roundtrips, although the spatio-spectral homogeneity is degraded due to mismatch of the MCS medium length after retuning. When *F*_therm_>30 cm, cavity retuning effectively restores the MCS performance. Representative beam profiles and temporal pulse evolution for *F*_therm_=20 cm are shown in Figs. S13b–c.

A similar compensation strategy applies if thermal lensing occurs predominantly at the cavity mirrors. Therefore, although thermal lensing perturbs the cavity degeneracy, the MCS condition can be robustly maintained through straightforward cavity retuning, making it compatible with high-average-power operation.


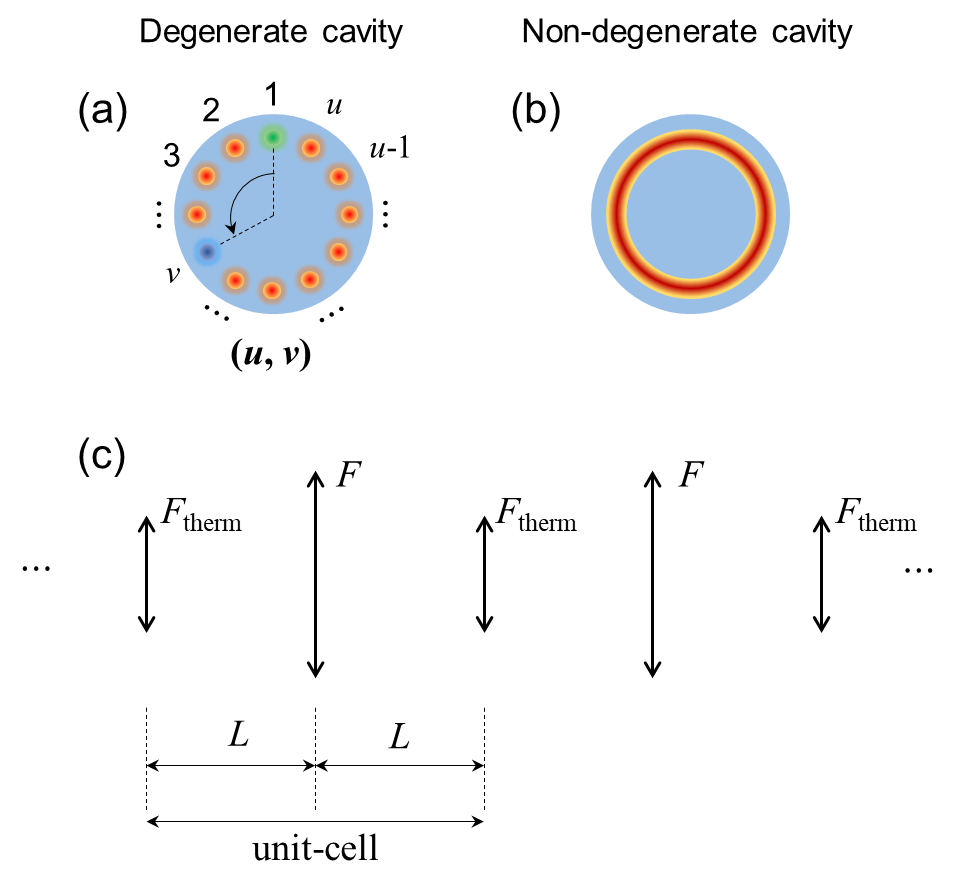


**Figure S12. (a)** Illustration of laser-spot distribution on a Herriott-type MPC with a degeneracy defined by indices (*u*, *v*). The green spot marks the initial position where the incident laser beam strikes, while the blue spot indicates its position after the first roundtrip. **(b)** Illustration of laser-spot distribution out of the degenerate condition. **(c)** Illustration of the equivalent lens-sequence representation of a linear cavity with the thermal lensing (*F*_therm_) considered.

**
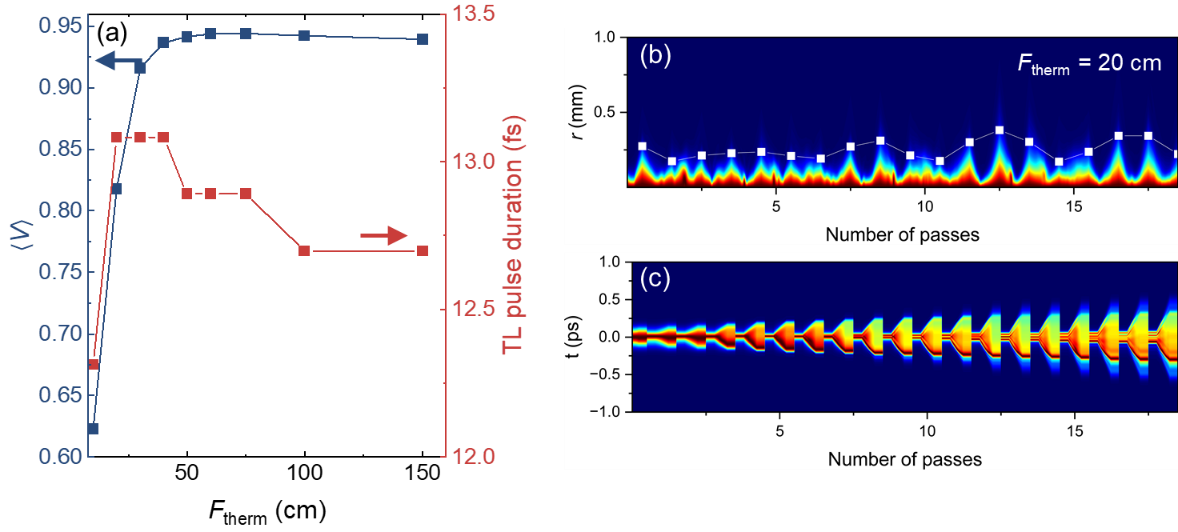
**

**Figure S13. (a)** Spatio-spectral homogeneity $\left\langle V \right\rangle$ and TL pulse duration as functions of thermal lensing focal length (*F*_therm_). **(b)** Beam-profile evolution and **(c)** temporal pulse evolution obtained from NLSE simulations for *F*_therm_=20 cm.

**S10. Supplementary Data**

**
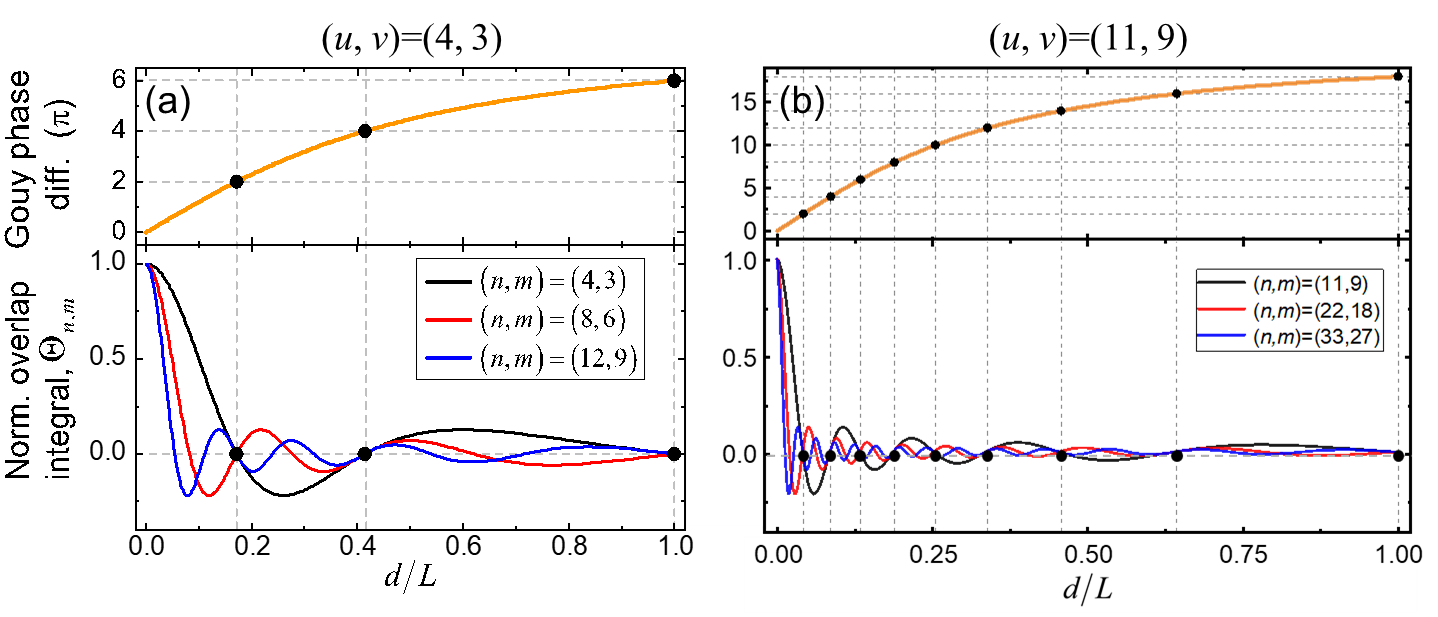
**

**Figure S14.** **(a)** Gouy phase difference accumulated over medium length 2*d* between the LG*_u_* and LG_0_ modes and corresponding normalized overlap integral Θ*_n_*_,_*_m_* as a function of *d*/*L*, calculated for and (*u*, *v*)=(4, 3). Dashed lines indicate medium lengths where the accumulated Gouy phase difference equals integer multiples of 2π, resulting in Θ*_n_*_,_*_m_* =0 for all degenerate modes. **(b)** Same as **(a)**, but for (*u*, *v*)=(11, 9)**.**


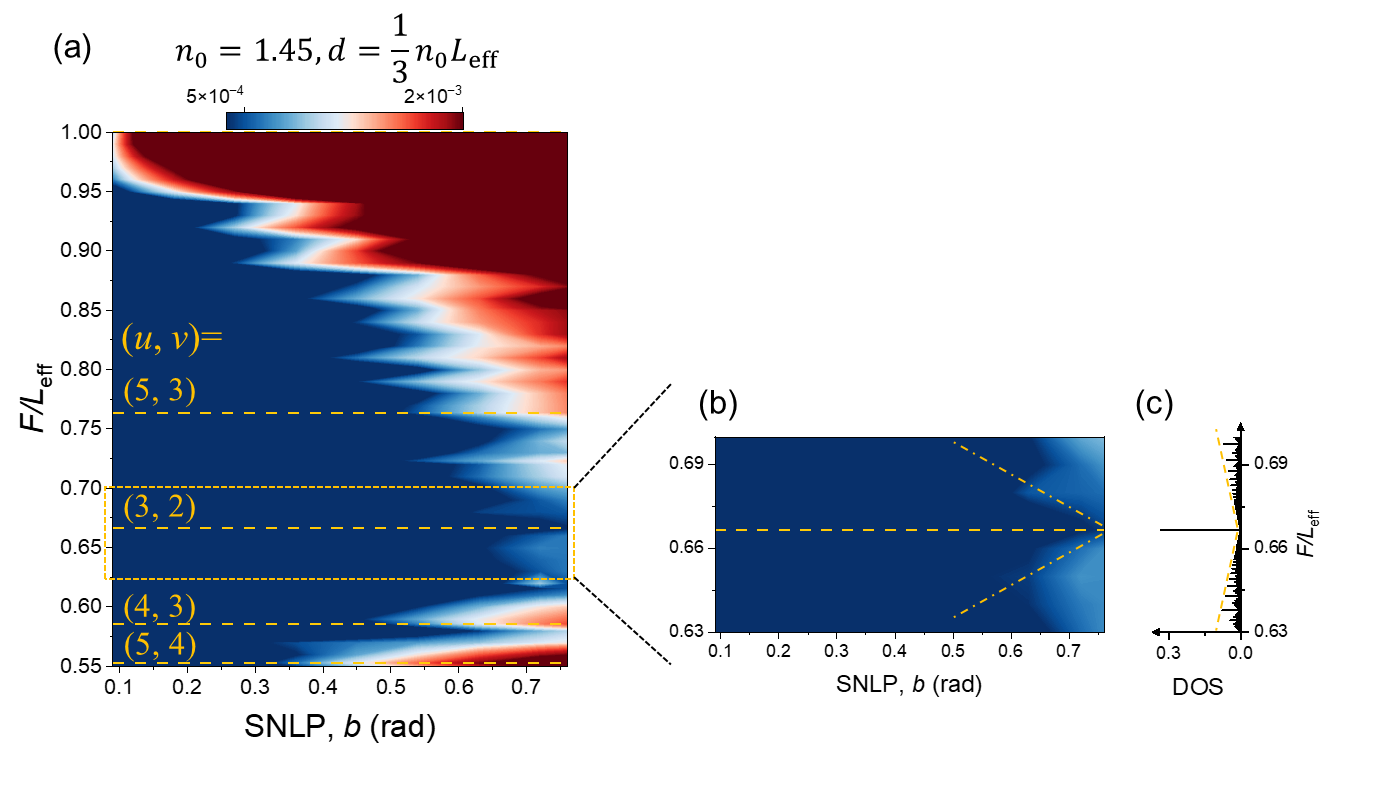


**Figure S15.** **(a)** Phase diagram of output beam inhomogeneity as a function of cavity geometry *F*/*L*_eff_ and SNLP *b*, for medium length corresponding to 2*d*= $\frac{2}{3}n_{0}L_{\mathrm{eff}}$. The refractive index is set to *n*_0_=1.45. Dashed lines indicate degeneracy geometries with (*u*, *v*)=(5, 3), (3, 2), (4, 3), and (5, 4). **(b)** Zoomed-in views of the phase diagrams near the degeneracy point (*u*, *v*)=(3, 2), as illustrated by the dashed-dotted boxes in **(a)**. **(c)** DOS results for the same *F*/*L*_eff_ region.

**References**

1. Vicentini, E. *et al.* Nonlinear pulse compression to 22 fs at 15.6 µJ by an all-solid-state multipass approach. *Opt. Express* **28**, 4541–4549 (2020).

2. Lavenu, L. *et al.* Nonlinear pulse compression based on a gas-filled multipass cell. *Opt. Lett.* **43**, 2252–2255 (2018).

3. Fox, A. G. & Li, T. Resonant Modes in a Maser Interferometer. *Bell Syst. Tech. J.* **40**, 453–488 (1961).

4. Zhang, S. *et al.* Solitary beam propagation in periodic layered Kerr media enables high-efficiency pulse compression and mode self-cleaning. *Light Sci. Appl.* **10**, 53 (2021).

5. Kevrekidis, P. G. *The Discrete Nonlinear Schrödinger Equation*. (Springer Berlin, Heidelberg, 2009).

6. Many Manda, B., Carretero-González, R., Kevrekidis, P. G. & Achilleos, V. Skin modes in a nonlinear Hatano-Nelson model. *Phys. Rev. B* **109**, 094308 (2024).
